# Emerging evidence for dysregulated proteome cargoes of tau-propagating extracellular vesicles driven by familial mutations of tau and presenilin

**DOI:** 10.20517/evcna.2023.44

**Published:** 2023-11-21

**Authors:** Vivian Hook, Sonia Podvin, Charles Mosier, Ben Boyarko, Laura Seyffert, Haley Stringer, Robert A. Rissman

**Affiliations:** ^1^Skaggs School of Pharmacy and Pharmaceutical Sciences, University of California, San Diego, CA 92093, USA.; ^2^Department of Neurosciences, University of California, San Diego, CA 92093, USA.; ^3^Veterans Affairs San Diego Health System, San Diego, CA 92093, USA.

**Keywords:** Tauopathies, mutant tau, mutant presenilin, exosome, extracellular vesicle, proteomics, Alzheimer’ s disease, frontotemporal dementia

## Abstract

Tau propagation, pathogenesis, and neurotoxicity are hallmarks of neurodegenerative diseases that result in cognitive impairment. Tau accumulates in Alzheimer’s disease (AD), frontotemporal dementia and parkinsonism linked to chromosome 17 (FTDP-17), chronic traumatic encephalopathy (CTE), progressive supranuclear palsy, and related tauopathies. Knowledge of the mechanisms for tau propagation in neurodegeneration is necessary for understanding the development of dementia. Exosomes, known as extracellular vesicles (EVs), have emerged as participants in promoting tau propagation. Recent findings show that EVs generated by neurons expressing familial mutations of tauopathies of FTDP-17 (P301L and V337M) (mTau) and presenilin (A246E) (mPS1) in AD induce tau propagation and accumulation after injection into rodent brain. To gain knowledge of the proteome cargoes of the mTau and mPS1 EVs that promote tau pathogenesis, this review compares the proteomes of these EVs, which results in important new questions concerning EV mechanisms of tau pathogenesis. Proteomics data show that EVs produced by mTau- and mPS1-expressing iPSC neurons share proteins involved in exocytosis and vesicle secretion and, notably, these EVs also possess differences in protein components of vesicle-mediated transport, extracellular functions, and cell adhesion. It will be important for future studies to gain an understanding of the breadth of familial genetic mutations of tau, presenilin, and other genes in promoting EV initiation of tau propagation and pathogenesis. Furthermore, elucidation of EV cargo components that mediate tau propagation will have potential as biomarkers and therapeutic strategies to ameliorate dementia of tauopathies.

## INTRODUCTION

Tau propagation and pathogenesis in the brain is a hallmark of tauopathy diseases of neurodegeneration that result in severe dementia. Tau accumulates in disorders of AD, FTDP-17, CTE, progressive supranuclear palsy, and related tauopathies^[1-4]^. Tauopathies are characterized by aggregation of hyperphosphorylated tau protein and neurofibrillary tangles (NFTs) in neurons^[2,3,5-7]^. Hyperphosphorylated tau loses its ability to interact with microtubules, which results in the destabilization of microtubules that compromise synaptic functions. Tau oligomers produce deficits in long-term potentiation and memory loss^[1-4,8-11]^. Knowledge of tau propagation mechanisms in brain neurodegeneration is necessary for understanding processes that control dementia. Advancements in defining mechanistic regulators of tau pathogenesis can provide targets for drug development to improve the health of tauopathy-afflicted patients.

Exosomes, a sub-category of extracellular vesicles (EV) which is referred to as EVs by the ISEV (International Society for Extracellular Vesicles)^[12]^, can mediate tau propagation in the brain in the context of dementias^[13-23]^. However, knowledge of the role of familial genetic mutations of tauopathies in EV-mediated spreading of tau has been limited. A search of the literature illustrates two studies by the Rissman and Hook groups that investigated tau-propagating EVs generated by neurons expressing the familial tau mutation (mTau) of P301L and V337M in FTP^[24]^, and the presenilin 1 mutation (mPS1) of A246E in AD^[25]^. Findings showed that these mTau and mPS1 mutations resulted in dysregulation of EV proteomes generated by human iPSC neurons expressing these mutations. To gain further understanding of the properties of the mTau and mPS1 EVs, this review addresses the question of whether these mutant EVs contain common proteome components and/or differences in their proteome composition. Results indicate that the mTau and mPS1 EVs contain shared proteins that are upregulated and downregulated, or present at similar levels in the two types of EVs. Notably, distinct proteins were observed in only mTau EVs, or only in mPS1 EVs. These data raise new questions concerning the breadth of familial mutations of tauopathies in generating EVs that initiate tau pathogenesis, and the mechanisms of EV cargo components responsible for promoting tau propagation. Such future EV research can lead to novel biomarkers and therapeutic strategies to reduce dementia that occurs in tauopathies.

**Figure 1:**
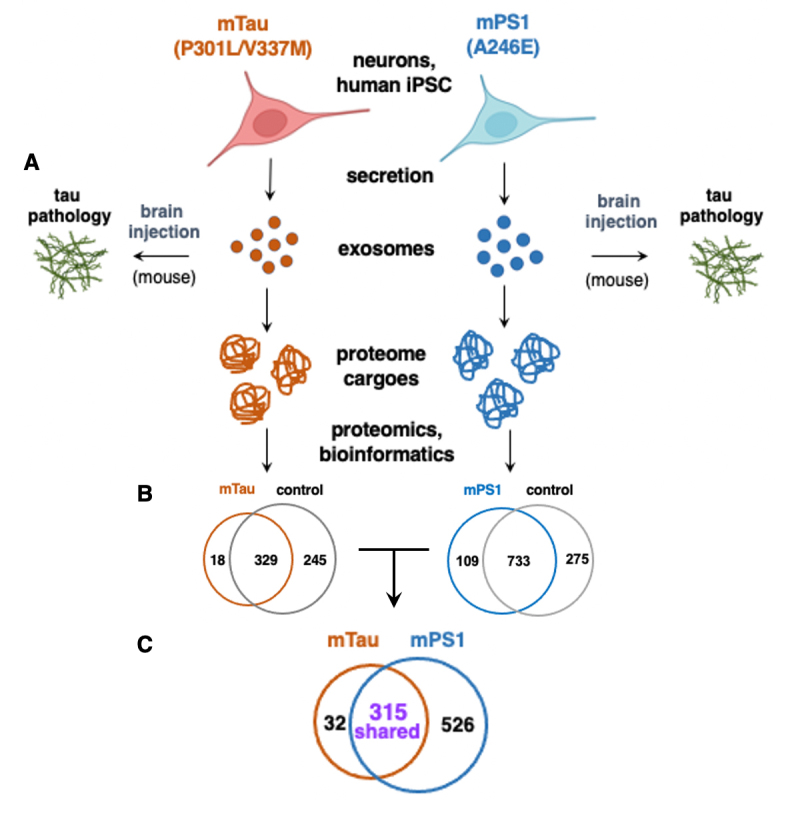
Analysis of proteome cargoes of tau-propagating EVs generated by neurons expressing familial mutant forms of tau and presenilin. EVs: extracellular vesicles. Tau-propagating EVs produced by human iPSC neurons expressing familial mutant tau P301L and V337 mutations of FTDP-17^[24]^, and mutant presenilin of A246E^[25]^ of Alzheimer’s disease were analyzed for (A) initiation of tau propagation in the rodent brain; (B) comparison of mutant EV proteome cargoes compared to wild-type control EVs; (C) comparison of mTau and mPS1 EVs proteomes that promote tau pathogenesis. The mTau iPSC neuronal cell line was obtained by lentivirus expression in a normal iPSC cell line, with a control consisting of expressing control lentivirus without the mutant Tau construct^[24]^. The mPS1 iPSC neuronal cell line was generated by reprogramming from a biopsy from a patient harboring the mPS1, and the control wild-type PS1 iPSC cell line was generated by reprogramming from a biopsy from a normal healthy patient having wild-type PS1^[25]^. It is noted that the mTau and mPS1 iPSC neurons are generated from different human patient biopsies and, therefore, possess different genetic backgrounds. The study of human iPSCs from different genetic backgrounds is logical to gain an understanding of tauopathies that afflict various human populations (The BioRender resource was used for the preparation of Figure 1).

**Familial tau and presenilin mutations to promote EV mediation of tau propagation in the brain.** EVs generated by neurons expressing familial mutations of tauopathies, human iPSC neurons expressing mTau with the P301L and V337M mutations of FTDP^[26,27]^, and mPS1 with the A246E mutation of AD^[28]^, were assessed for initiation of tau propagation in mouse brain and were subjected to proteomics and bioinformatics to analyze proteome cargoes [Figure 1].

Expression of mutant tau (mTau) with the P301L and V337M mutations^[26,27]^ in human iPSC neurons resulted in the accumulation of intracellular NFTs^[29]^. EVs secreted from these neurons induce tau aggregates and neurotoxicity in normal recipient human iPSC neurons^[29]^, and injections of the mTau EVs into mouse brain result in tau propagation^[29,30]^.

With respect to a familial AD mutation, EVs generated by iPSC neurons with the presenilin (PS1) mutation of A246E^[28]^ were capable of initiating tau propagation and aggregation in the mouse brain hippocampus upon intracranial injection^[31]^. These findings provide evidence that familial mutations of tauopathies and AD may generate EVs that propagate tau pathogenesis *in vivo* when introduced into rodent brains.

## MUTANT TAU AND MUTANT PS1 GENERATE DYSREGULATED EV PROTEOME CARGOES

**mTau EVs possess dysregulated proteome cargoes.** Assessment of the protein cargo of EVs generated by human iPSC neurons expressing mTau (mutations P301L and V337M) compared to wild-type (Wt) Tau, was conducted by proteomics and bioinformatics analysis^[24]^. The mTau EVs displayed dysregulated protein cargoes compared to control EVs [Figure 1B]. Assessment of the mTau and control EVs found (1) proteins uniquely observed in only mTau EVs; (2) proteins found in only control EVs; (3) shared proteins in mTau and controls that were upregulated or downregulated.

The mTau EVs uniquely contained ANP32A (also known as I1PP2A) that was not present in controls. ANP32A inhibits PP2A phosphatase which regulates the phosphorylation state of p-Tau^[32]^. ANP32A is upregulated in AD brain^[32]^. Downregulation of ANP32A in tau transgenic mice resulted in the rescue of memory deficits, amelioration of synaptic dysfunction, and attenuation of AD-like tau hyperphosphorylation^[33]^.

The mTau EVs lacked numerous proteins present in only controls that participate in pathways of localization, vesicle transport, and protein binding functions. Proteins common to mTau and controls possess EV functions of vesicle-mediated transport, exocytosis, and secretion processes. These findings indicate mTau as a regulator of the proteome cargo of EVs that is capable of inducing tau propagation in the brain.

**mPS1 EVs harbor dysregulated proteome components, including regulators of p-tau**. EVs generated by human iPSC neurons with mPS1 of A246E were compared to those produced by wt neurons^[25]^. The mPS1 and control wt EVs showed (1) proteins observed in only mPS1 EVs; (2) proteins found in only control EVs; (3) shared proteins in mPS1 and controls that were upregulated or downregulated [Figure 1B]. The mPS1 mutation resulted in EV cargo with the acquisition of extracellular matrix and protease proteins, and the reduction of proteins involved in protein translation with proteasome functions^[25]^. The mPS1 EVs possessed changes in protein phosphatases and kinases that regulate p-tau^[25]^ [Supplementary Table 1]. Notably, the mPS1 EVs lacked PPP2R2, found only in control EVs, which is a regulatory subunit of protein phosphatase 2A. PPP2R2A targets p-tau as the substrate for dephosphorylation^[34]^. In addition, the phosphatase catalytic subunits of PPP1CA, PPP1CB, and PPP3CA were absent in mPS1 EVs, and were found only in the control EVs. The loss of PPP2R2A and several catalytic subunits of protein phosphatases may reduce dephosphorylation that would increase p-tau involved in tau pathology.

With respect to protein kinases involved in tau phosphorylation, the PRKDC, CSKN2, and CDK1 kinases were absent in the mPS1 EVs, and were present in only control EVs^[25]^. FYN, a tyrosine kinase, was upregulated in mPS1 compared to control EVs; FYN has dual functions of direct phosphorylation of tau and inhibition of PP2A that dephosphorylates tau^[35]^. The MAPK3 and MAPK1 kinases were moderately downregulated in mPS1 compared to control EVs. These results indicate dysregulation of the spectrum of several tau protein kinases in the mPS1 EVs [Supplementary Table 1].

These proteomics data demonstrate dysregulation of mTau and mPS1 EV proteome cargoes that mediate tau-propagation^[24,25]^.

## COMPARISION OF mTAU AND mPS1 EV PROTEOME CARGOES REVEALS SIMILARITIES AND DIFFERENCES

**Direct comparison of mTau and mPS1 EV proteome cargoes.** Since the mTau and mPS1 EVs both propagate tau spreading^[29-31]^, it is of interest to assess their shared and distinct proteins. Proteomics analysis revealed similar and different cargo components of mTau and mPS1 EVs [Figure 1C]. A comparison of mTau and mPS1 EV proteomes found 315 proteins that were shared by both types of EVs, 32 proteins that were found only in the mTau EVs, and 526 proteins that were found in only the mPS1 EVs [Figure 1C]. These data indicate shared and distinct proteome components in mTau and mPS1 EVs.

**Figure 2:**
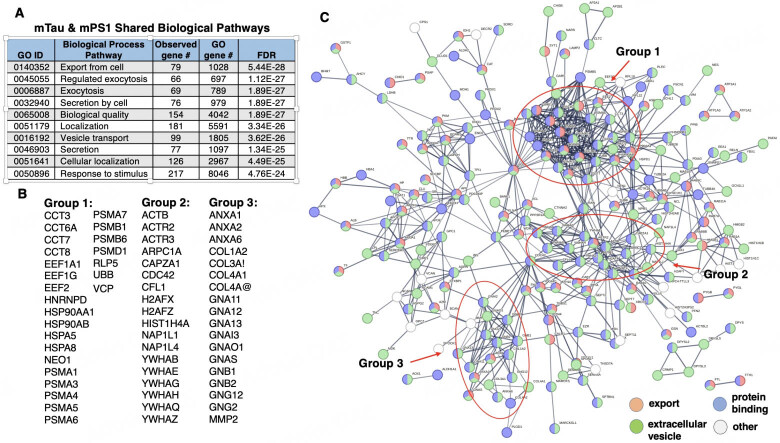
Protein network analysis of proteins shared by mTau and mPS1 EVs. (A) Shared biological pathways of mTau and mPS1 EV proteomes. Shared proteins of mTau and mTPS1 EVs^[24,25]^ were assessed for functional biological pathways by GO analysis using FDR significance levels of < 0.05. In fact, highly significant FDRs are indicated of 10^-24^ to 10^-28^; (B) Hub proteins of interaction networks. Protein components of hubs of protein interaction networks of groups 1-3 are listed. Functions of these proteins are provided in Supplementary Table 2; (C) STRING-db protein interaction networks of proteins shared by mTau and mPS1 EVs. Interaction utilized scores set to high confidence (0.7 on a scale of 0-1) that predicted interactions exist among the proteins illustrated. EVs: extracellular vesicles.

**Shared proteome components in mTau and mPS1 EVs assessed in system protein networks.** Gene ontology (GO) analysis of proteins shared by mTau and mPS1 EVs illustrated significant similarity in biological pathways of cellular export and exocytosis, vesicle transport and secretion, and response to stimuli [Figure 2A]. These EVs also showed similar molecular binding pathways involving proteins, anions, adhesion, carbohydrates, small molecules, and RNA. STRING analysis of shared mTau and mPS1 proteins indicated a complex network of protein interaction systems, including cell export, extracellular vesicles, and protein binding [Figure 2B and C]. Three groups of hubs were observed in these networks consisting of functional areas of (1) chaperones including heat-shock proteins, and proteasome subunits; (2) actin-related proteins, nuclear histones and nucleosome components, and tyrosine-monooxygenase related proteins; (3) annexins, collagens, and G-protein subunits [Supplementary Table 2]. These network functions indicated shared protein functions of the mTau and mPS1 EVs.

Proteins present at similar levels in mTau compared to mPS1 EVs possess functions of secretion and vesicular trafficking, chaperones and protein folding, lysosomes, cell viability and migration, transcription, and signal transduction^[24,25]^ [Supplementary Table 3].

**Figure 3:**
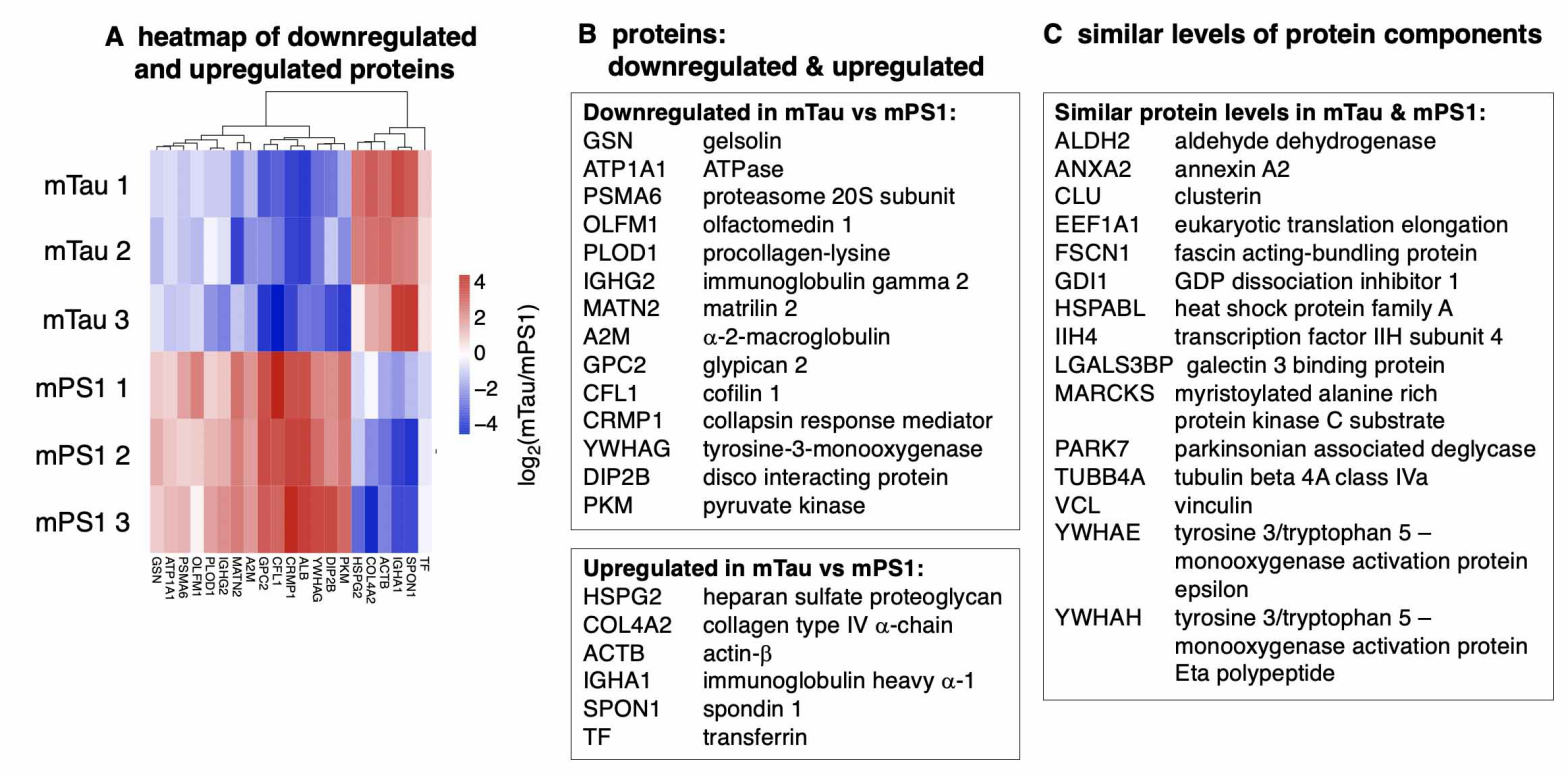
Proteins that are downregulated, upregulated, or of similar levels in mTau compared to mPS1 EVs. (A) Heatmap with hierarchical clustering illustrates downregulated and upregulated proteins in mTau compared to mPS1 EVs. The heatmap shows log_2_(mTau/mPS1) ratios that were significant (*P* < 0.05); (B) Downregulated and upregulated proteins in mTau *vs*. mPS1 EVs. Protein components found to be significantly downregulated or upregulated in mTau compared to mPS1 proteomes are listed by gene and protein names. Significance is defined as *P* < 0.05 for log_2_ ratios of (mTau/mPS1) quantitation of proteins. Functions of these proteins are provided in Supplementary Table 4; (C) Proteins at similar levels in mTau and mPS1 EVs. Proteins present in both mTau and mPS1 EVs of similar levels are listed by gene names. EVs: extracellular vesicles.

Upregulated and downregulated proteins shared by mTau and mPS1 EVs were assessed by log_2_(mTau/mPS1) ratios, illustrated by a heatmap and hierarchical clustering [Figure 3]. Downregulated proteins in mTau compared to mPS1 EVs consisted of proteins of gelsolin and cofilin for actin binding, proteasome and alpha-2-macroglobulin protease inhibitor for proteolysis, ATPase Na^+^/K^+^ transporting subunit, olfactomedin involved in neural crest cell production, procollagen deoxygenase for collagen modification, immunoglobulin heavy constant gamma 2 isotype, matrilin extracellular matrix protein, glypican proteoglycan involved in signaling, collapsin response mediator, albumin, tyrosine 3-monooxygenase, disco interacting protein for transcriptional regulation, and pyruvate kinase of the glycolytic pathway [Supplementary Table 4]. Upregulated proteins in mTau compared to mPS1 consisted of heparan sulfate proteoglycan, collagen type IV alpha chain, actin beta, immunoglobulin heavy constant alpha-1 isotype, spondin extracellular matrix protein, and transferrin for iron transport [Supplementary Table 4]. These shared proteins include functions of structural binding proteins, proteolysis, collagen and extracellular matrix support proteins, immunoglobulins, and metabolism [Supplementary Table 4].

**Figure 4:**
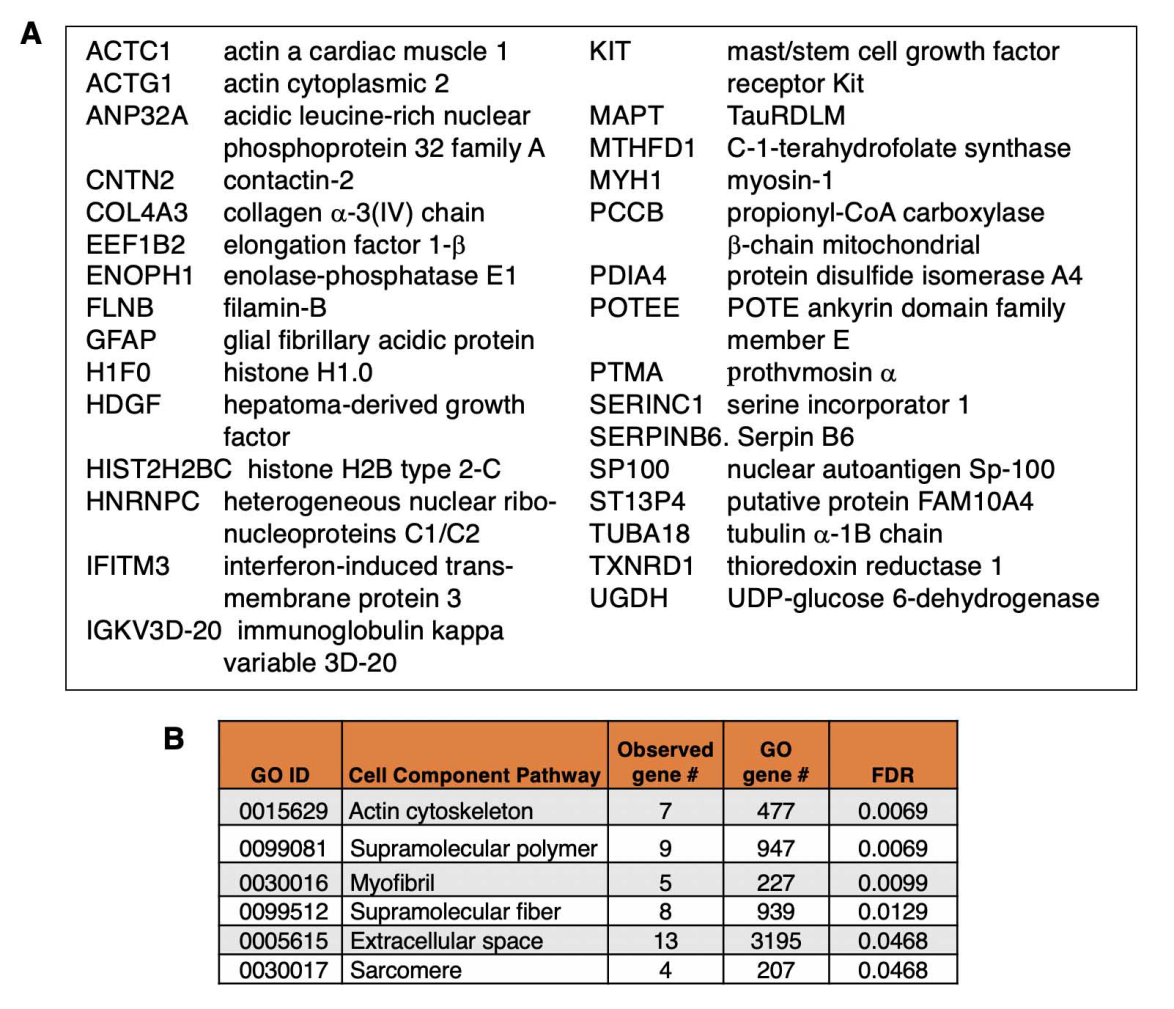
Proteins present in only mTau EVs (not mPS1 EVs). (A) Proteins present in only mTau EVs (not mPS1 EVs). Such proteins are listed by their gene symbol names; (B) GO analysis of proteins in only mTau EVs. GO analysis of proteins present in only the mTau EVs (and not in the mPS1 EVs) indicated significant cell component pathways (FDR < 0.05). EVs: extracellular vesicles.

**Distinct proteins found in only mTau or only in mPS1 EVs**. The mTau EV proteome contained 32 distinct proteins that were not present in mPS1 EVs [Figure 1C]. These distinct mTau EV proteins represented GO pathways of structural actin, myofibril, and related [Figure 4].

**Figure 5:**
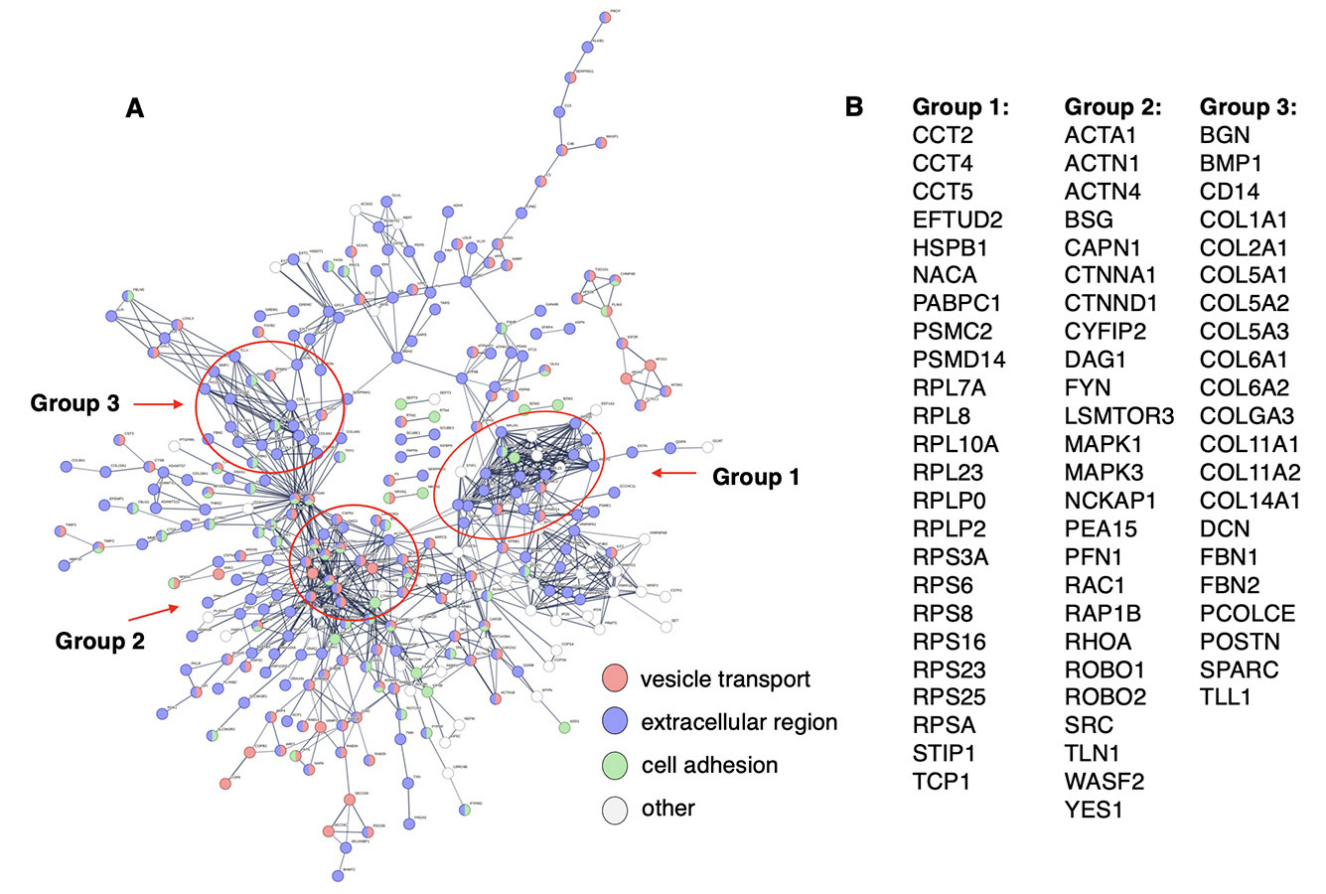
Protein network of proteins present in only mPS1 EVs (not mTau EVs). (A) STRING-db protein interaction networks of proteins present in only mPS1 EVs. Interactions utilized scores set to high confidence that predicted interactions exist among the proteins illustrated; (B) Hub proteins of interaction networks. Protein components of three hubs of protein interaction networks are illustrated. Functions of proteins are provided in Supplementary Table 5.

The proteome of the mPS1 EVs contained 526 proteins that were not observed in the mTau EVs [Figure 1C]. STRING analysis of these unique mPS1 EV components illustrated protein interaction pathways of vesicle-mediated transport, extracellular functions, cell adhesion, and other functions [Figure 5]. Three groups of interaction network hubs were observed consisting of (1) proteins functioning as chaperones, proteasome proteolysis, and ribosomal proteins; (2) actin proteins, calpain proteolysis, cell adhesion, kinases, ras, and oncogene proteins; (3) collagens and extracellular matrix proteins [Figure 5 and Supplementary Table 5]. These findings show that the mPS1 EVs contain proteins for EV functions that differ from those found in the mTau EVs.

Overall, these data indicate that mechanisms of mTau and mPS1 EV-mediated tau propagation may involve (1) shared proteins; (2) distinct proteins present in each EV type; (3) shared and differential proteins for modified proteomes. Future investigation of these possible mechanisms will be of interest to address the responsible EV proteins that promote tau propagation.

## FUTURE INVESTIGATION OF TAU-PROPAGATING EVs DERIVED FROM NEURONS EXPRESSING FAMILIAL TAU AND PRESENILIN MUTATIONS OF TAUOPATHIES TO ELUCIDATE TAU-PROPAGATING MECHANISMS

**Distinct and similar proteins of EV proteomes in tau pathogenesis**. The findings of similar and different proteins of the EV cargoes of mTau and mPS1 raise several questions about the mechanisms of tau propagation. An important question to assess is whether common proteins, different proteins, or a combination of shared and distinct proteins participate in mTau and mPS1 EV-mediated tau propagation. The mTau EV proteome consisted of 347 proteins, of which 315 are common to those of the mPS1 EVs. In addition, mTau EVs had 32 distinct proteins that differed from mPS1 EVs. While the mPS1 EVs shared 315 proteins with mTau, mPS1 EVs contained 526 distinct proteins that were not found in mTau EVs. Elucidation of mTau and mPS1 EV proteins participating in tau propagation will be significant in future studies. Elucidation of EV proteins involved in tau pathogenesis may include, for example, a gene silence screening approach to reduce specific EV proteins to prevent EV-mediated transcellular propagation of tau aggregates in neuronal cultures. Other approaches to elucidate responsible EV proteins for tau propagation may be developed by investigators in the field.

**Figure 6:**
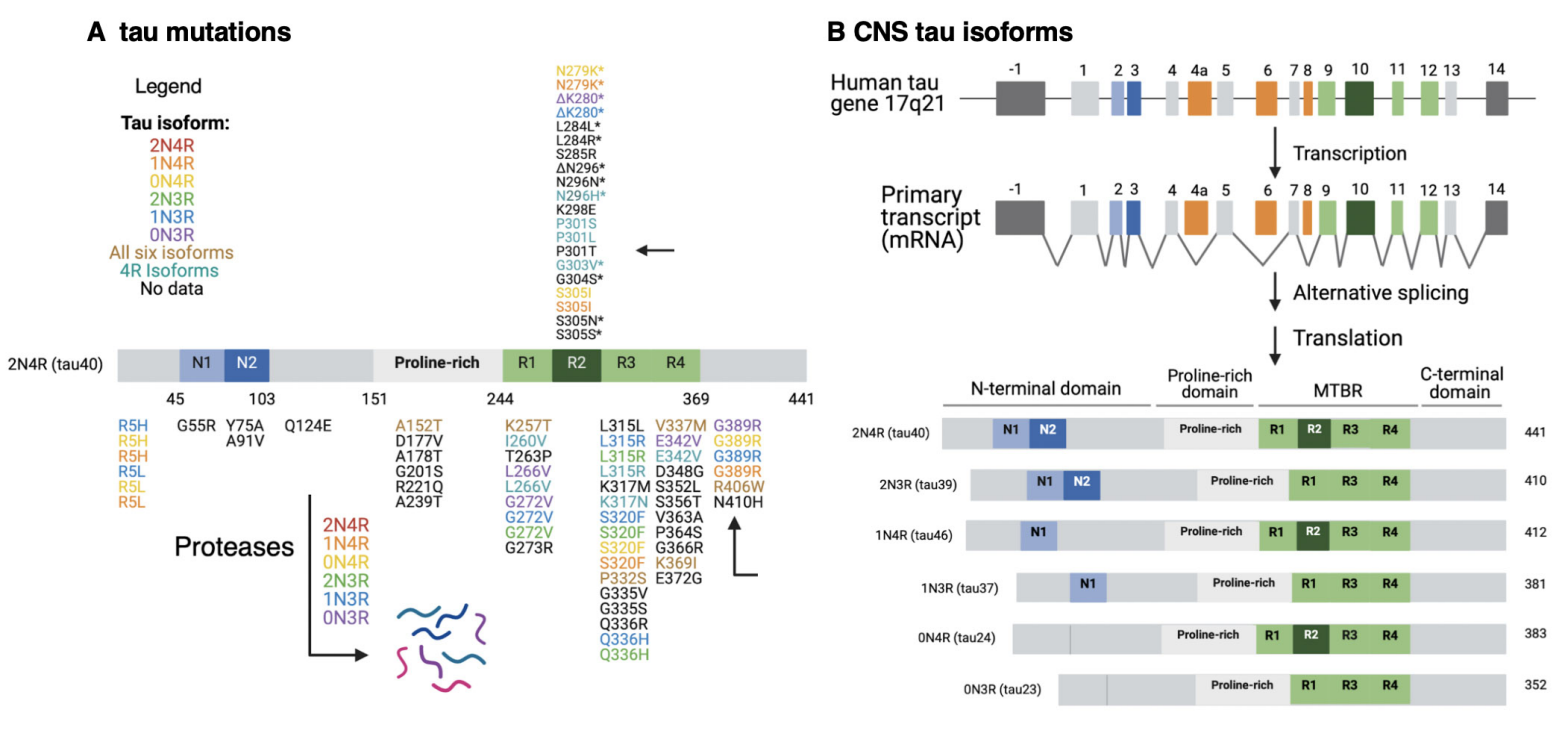
Tau mutations and tau protein isoforms. (A) Tau mutations. Tau missense and deletion mutations located in six tau isoforms (listed in legend for tau isoforms) are illustrated. The mutations are mapped for the representative 2N4R isoform; (B) Tau protein isoforms. Six CNS tau isoforms are illustrated with respect to the N1 and N2 domains at the N-terminal regions with the four R1, R2, R3, and R4 domains of the MTBR. The six tau isoforms are shown as 2N4R, 2N3R, 1N4R, 1N3R, 0N4R, and 0N3R. MTBR: microtubule-binding domain region.

**Figure 7:**
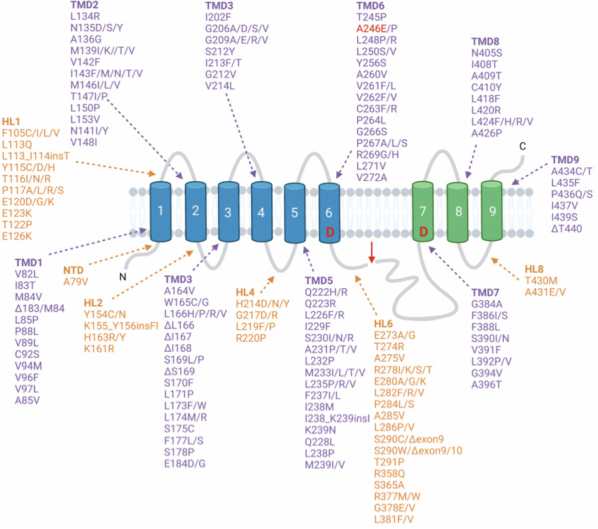
PS1 mutations (PS1: presenilin 1). Presenilin 1 is one of the four catalytic subunits of the γ-secretase protein complex^[50]^. Over 300 PS1 mutations have been identified, covering ~ 25% of the PS1 residues, which account for the majority of FAD mutations^[40]^. Most are missense mutations that localize in the TMDs and in the HLs. Upon assembly and maturation of the complex, presenilin 1 is cleaved within the large cytoplasmic loop into two fragments, the NFT comprising of TMDs 1-6 (blue) and the CTF comprising TMDs 7-9^[51]^. Cleavage occurs between the two aspartate active site residues in TMDs 6 and 7 (labeled D)^[52]^. The FAD mutation A2456 generated for the iPSC neurons in the mPS1 study^[24,25]^ is shown in red. FAD: familial AD; TMDs: transmembrane domains; HLs: hydrophilic loops; NFT: N-terminal fragment; CTF: C-terminal fragment.

**Familial mutations of tauopathies and involvement of EV facilitation of tau propagation**. Another question to assess is whether other familial mutations of Tau and PS1, as well as other genetic mutations, in tauopathies result in neuronal production of EVs that promote tau propagation. A multitude of Tau mutations [Figure 6A] exist in six Tau protein isoforms in tauopathies^[3,36-38]^ [Figure 6B] and numerous PS1 mutations exist in Alzheimer’s disease^[39-41]^ [Figure 7]. It will be important to gain an understanding of the roles of numerous familial forms of mutant Tau and mutant PS1, as well as other familial mutations, in the production of tau-propagating EVs.

**Proteolytic tau fragments involved in EV-mediated tau propagation**. Furthermore, an unanswered question to address is what tau isoforms and neurotoxic proteolytic fragments are induced by mTau or mPS1 EVs? In the brain, the tau transcript undergoes alternative splicing to generate six tau isoforms that vary in repeated microtubule-binding domains (R1, R2, R3, R4) and variant N-terminal domains^[3,36-38]^ [Figure 6B]. These tau isoforms undergo proteolysis to generate neurotoxic tau fragments. The cleavage of tau into N-terminal and C-terminal proteolytic fragments by proteases, including caspases, calpains, and cathepsins, exacerbates tau aggregation and is closely related to pathological transmission throughout the brain^[36]^. It will be important to gain an understanding of what tau isoforms and proteolytic fragments participate in mTau and mPS1 EV-mediated tau pathogenesis.

**Mechanisms of EV-mediated tau pathogenesis in target cells.** A further question to address is how EVs promote tau spreading in target cells involving EV proteins and target cell systems. Several studies found that EVs are internalized within intracellular endosomes which fuse with lysosomes, and the EVs then induce lysosomal permeabilization that allows EV tau to escape into the cytosol to promote tau aggregation^[42,43]^. Cytosolic tau can also activate the inflammasome that induces IL-1β production^[44]^. It will be important for future studies to elucidate the primary mechanisms of tau pathogenesis mediated by dysregulated mutant EVs.

## FUTURE PERSPECTIVES FOR CLINICAL BIOMARKER AND THERAPEUTIC STRATEGIES TO BLOCK EV-MEDIATED TAU PROPAGATION

The conclusions that can be drawn from these studies are that there are similarities and differences in the proteome components of the mTau and mPS1 EVs. It will be of interest to investigate whether common proteins present in both mTau and mPS1 EVs participate in tau propagation. Furthermore, differences in proteome components of these mutant EVs raise the question of different properties of tau pathogenesis induced by the mTau compared to the mPS1 EVs. The EV proteome components provide the potential for biomarkers for tau-propagating EVs, and/or for specific markers of EVs resulting from particular familial mutations of tauopathies. Selective biomarkers of EVs representing familial mutation subtypes may be useful in future clinical biomarkers and therapeutic strategies to modulate EV-mediated tau propagation.

Neuronal^[13,45]^ as well as astrocyte^[46-48]^ and microglial-derived^[49]^ EVs have been studied from biobanked clinical trial samples and provide the potential for application as biomarkers of clinical familial tauopathies. EVs from neurons and glia cells can exit the brain and reach the plasma^[30,46-49]^ for clinical analysis. It will, therefore, be of interest to compare EVs generated by human brain neurons or glia in familial tauopathy diseases to those in plasma to assess the potential for plasma EVs to represent biomarkers of brain EV-mediated tau pathogenesis.

Elucidation of mechanisms involved in EV-mediated tau propagation can lead to new drug targets to block tau spreading and toxicity in the brain. Drug targeting of molecular components that participate in EV induction of tau pathogenesis may lead to future new therapeutic strategies to reduce dementia of tauopathies.

## DECLARATIONS

### Availability of data and materials

Mass spectrometry LC-MS/MS data files can be accessed at 222.proteomexchange.org under the dataset identifier number PDX016101 for the mTau EV proteomics data, and number PXD019424 for the mPS1 EV proteomics data.

### Copyright

© The Author(s) 2023.
