# Supplementary material for: Emerging evidence for dysregulated proteome cargoes of tau-propagating extracellular vesicles driven by familial mutations of tau and presenilin
Source: Extracell Vesicles Circ Nucl Acids. 2023 Nov 21;4(4):588–98. doi: 10.20517/evcna.2023.44 (PMC10732590; doi:10.20517/evcna.2023.44)
Supplement: Supplementary file 1 [file evcna-4-4-588-SupplementaryMaterials.pdf]

Supplemental Information

Emerging Evidence for Dysregulated Proteome Cargoes of Tau-Propagating Extracellular Vesicles Driven by Familial Mutations of Tau and Presenilin

Vivian Hook[1,2*], Sonia Podvin[1], Charles Mosier[1], Ben Boyarko[1], Laura Seyffert[1], Haley Stringer[1], and Robert A. Rissman[2,3]

[1]Skaggs School of Pharmacy and Pharmaceutical Sciences, University of California, San Diego, La Jolla, CA 92093, USA.
[2]Department of Neurosciences, University of California San Diego, La Jolla, CA 92093, USA.
[3]Veterans Affairs San Diego Health System, La Jolla, CA 92093, USA.

Correspondence to: Dr. Vivian Hook, Skaggs School of Pharmacy and Pharmaceutical Sciences, University of California, 9500 Gilman Dr. MC0657, La Jolla, San Diego, CA 92093, USA. E-mail: vhook@ucsd.edu

Supplementary Tables:

Supplementary Table 1. Tau Phosphatases and kinases in mPS1 compared to control EVs … p. 2-4

Supplementary Table 2. Hub proteins of shared mTau and mPS1 interaction networks …………p. 5-14

Supplementary Table 3. Functions of proteins present at similar levels in mTau and mPS1 EVs …………… p.15-17

Supplementary Table 4. Downregulated and upregulated proteins in mTau compared to mPS1 EVs ………… p.18-23

Supplementary Table 5. Hub proteins present in only mPS1 exosomes, and not in mTau EVs …p. 24-38

Supplementary Table 1. Tau Phosphatases and Kinases in mPS1 Compared to Control EVs

The proteome content of mPS1 exosomes (derived from human iPSC neurons expressing the familial mutation of PS1 (A246E) was compared to control exosomes (from iPSC neurons expressing wild-type PS1) [14]. Proteome components of phosphatases and kinases involved in regulating the phosphorylation status of tau are compared with respect to mPS1 and control exosome cargoes.

Gene name	Description	mPS1 or control exosomes			Role in tau phosphorylation	References
		Only mPS1	Only control	Shared, Log_2 mPS1/cntrl		
**Phosphatases:**						
**PPP1CA**	serine/threonine protein phosphatase PP1-alpha catalytic subunit		+		PP1 α catalytic subunit contributes to de-phosphorylation of tau	29-31
**PPP1CB**	serine/threonine protein phosphatase PP1-beta catalytic subunit		+		PP1 β catalytic subunit contributes to de-phosphorylation of Tau	29-31
**PPP3CA** (calcineurin)	serine/threonine-protein phosphatase PP2B catalytic subunit		+		Calcineurin de-phosphorylates pS262 and pS396 on Tau which are both found in parahelical filaments.	29-31
**PPP2R2A**	serine/threonine protein phosphatase 2A 55 kDa regulatory subunit B alpha		+		The B55α regulatory subunit of PP2A recognizes p-Tau substrate for PP2A de-phosphorylation.	29-36
**PPP2R1A**	serine/threonine-protein phosphatase 2A 65 kDa regulatory subunit A alpha			+ 1.670	This subunit of PP2A is required for Tau (and other substrate) de-phosphorylation	29-36
**Kinases:**						
**PRKDC**	DNA-dependent protein kinase catalytic subunit		+		DNA-PK catalytic subunit phosphorylates Tau *in vitro*	37
**CSNK2B**	casein kinase II subunit beta		+		CK2 phosphorylates SET to induce PP2A inhibition resulting in hyper-phosphorylation of tau.	36
**CDK1**	cyclin-dependent kinase 1		+		CDK has high affinity for phosphorylation of SP motifs of tau.	39, 40
**FYN**	tyrosine protein kinase Fyn			+ 0.131	Fyn phosphorylates tau and also inhibits the main Tau phosphatase PP2A by phosphorylation	41-43
**MAPK3** (ERK1)	mitogen-activated protein kinase 3			+ -0.156	Erk1 is involved in abnormal Tau phosphorylation in AD brains.	41, 44
**MAPK1** (ERK2)	mitogen-activated protein kinase 1			+ -0.768	Erk2 phosphorylates tau at 15 sites *in vitro* and can contribute to tauopathy.	41, 44, 45
**SRC**	proto-oncogene tyrosine-protein kinase Src			+ na	Src phosphorylates Tau present in neurofibrillary tangles	42

Supplementary Table 2. Hub proteins of shared mTau and mPS1 interaction networks

Protein interaction networks of proteins shared by mTau and mPS1 exosomes are illustrated in Figure 2, which shows three main hubs of proteins. Protein components of these three hubs are illustrated in this Table to indicate the protein names of the indicated gene, its normal function, and examples of findings in the field for functions related to Alzheimer's disease (AD) or related tauopathies.

Hub Group	Gene name	Protein name	Normal functions	Functions related to AD or tauopathies
**Group 1**				
	CCT3	Chaperonin Containing TCP1 Subunit 3	Component of chaperonin-containing T-complex, assists folding of proteins. Telomere maintenance.	--
	CCT6A	Chaperonin Containing TCP1 Subunit 6A	Component of chaperonin-containing T-complex, assists folding of proteins. Telomere maintenance.	--
	CCT7	Chaperonin Containing TCP1 Subunit 7	Component of chaperonin-containing T-complex, assists folding of proteins. Telomere maintenance.	--
	CCT8	Chaperonin Containing TCP1 Subunit 8	Component of chaperonin-containing T-complex, assists folding of proteins. Telomere maintenance.	--
	EEF1A1	Eukaryotic Translation Elongation Factor 1 Alpha 1	Subunit of elongation factor-1 complex, Promotes GTP-dependent binding of aminoacyl-tRNA to A-site of ribosomes during protein biosynthesis.	Expression reduced in hippocampus in AD patients [1]
	EEF1G	Eukaryotic Translation Elongation Factor 1 Gamma	Subunit of elongation factor-1 complex, Probably plays a role in anchoring the complex to other cellular components.	--
	EEF2	Eukaryotic Translation Elongation Factor 2	Promotes the GTP-dependent translocation of the nascent protein chain from the A-site to the P-site of the ribosome	Potential biomarker for AD [2] Levels of eEF2 decreased in AD brain. Relationship with Tau [3]
	HNRNPD	Heterogeneous Nuclear Ribonucleoprotein D	Binds to nucleic acids, complexes with nuclear RNA. Influence pre-mRNA processing, mRNA metabolism and transport.	--
	HSP90AA1	Heat Shock Protein 90 Alpha Family Class A Member 1	Aids in the proper folding of specific target proteins by use of an ATPase activity that is modulated by co-chaperones. Cell cycle control and signal transduction.	Expression of HSP90 significantly higher in cells from AD patients, patient derived cell lines with PS1 mutations. [4] Involved in protein-protein interactions in AD and dementia. [5]

	HSP90AB1	Heat Shock Protein 90 Alpha Family Class B Member 1	Involved in signal transduction, protein folding and degradation and morphological evolution. Constitutive form of the cytosolic 90 kDa heat-shock protein and is thought to play a role in gastric apoptosis and inflammation.	Downregulated in non-AD astrocytes, coexpressed with tau and astrocytes in AD samples. Neurodegeneration and astrogliosis related to hsp90ab1 in AD. [6]<br>High expression in brain in individuals with neurodegenerative disorders. [7]
	HSPA5 (Grp78)	Heat Shock Protein Family A (Hsp70) Member 5	ER chaperone that plays a key role in protein folding and quality control in the endoplasmic reticulum lumen	Upregulation, colocalization with Abeta and accumulation in accumulates in plaques in brain tissue of AD patients. [8]
	HSPA8 (Hsc70)	Heat Shock Protein Family A (Hsp70) Member 8	Functions as a chaperone, and binds to nascent polypeptides to facilitate correct folding. It also functions as an ATPase in the disassembly of clathrin-coated vesicles during transport of membrane components.	Tau-binding CHIP-**Hsc70** complex responsible for tau clearance. [9]
	NEO1	Neogenin 1	Cell surface receptor regulating cell adhesion in many diverse developmental processes, including neural tube and mammary gland formation, myogenesis and angiogenesis	--
	PSMA1	Proteasome 20S Subunit Alpha 1	Component of the 20S core proteasome complex involved in the proteolytic degradation of most intracellular protein.	--
	PSMA3	Proteasome 20S Subunit Alpha 3	Component of the 20S core proteasome complex involved in the proteolytic degradation of most intracellular proteins	May be a co-diagnostic effector gene for AD. [10]
	PSMA4	Proteasome 20S Subunit Alpha 4	Component of the 20S core proteasome complex involved in the proteolytic degradation of most intracellular proteins	Decreased expression level in patients with AD. [11]
	PSMA5	Proteasome 20S Subunit Alpha 5	Component of the 20S core proteasome complex involved in the proteolytic degradation of most intracellular proteins	Downregulation of PSMA5 may play a role in APP overexpression-induced proliferation impairment. [12]
	PSMA6	Proteasome 20S Subunit Alpha 6	Component of the 20S core proteasome complex involved in the proteolytic degradation of most intracellular proteins	Potential biomarker for AD, identified as a hub gene in female human with AD. [13]
	PSMA7	Proteasome 20S Subunit Alpha 7	Component of the 20S core proteasome complex involved in the	--

			proteolytic degradation of most intracellular proteins	
	PSMB1	Proteasome 20S Subunit Beta 1	Non-catalytic component of the 20S core proteasome complex involved in the proteolytic degradation of most intracellular proteins.	--
	PSMB6	Proteasome 20S Subunit Beta 6	Catalytic component, involved in the proteolytic degradation of most intracellular proteins. This complex plays numerous essential roles within the cell by associating with different regulatory particles. Associated with two 19S regulatory particles, forms the 26S proteasome and thus participates in the ATP-dependent degradation of ubiquitinated proteins	--
	PSMD1	Proteasome 26S Subunit, Non-ATPase 1	Component of the 26S proteasome, a multiprotein complex involved in the ATP-dependent degradation of ubiquitinated proteins. Plays role in the maintenance of protein homeostasis by removing misfolded or damaged proteins.	Identified as an AD immune-related hub gene. [14]
	RPL5	Ribosomal Protein L5	Component of the ribosome, a large ribonucleoprotein complex responsible for the synthesis of proteins in the cell.	--
	UBB	Ubiquitin B	Ubiquitin has a major role in targeting cellular proteins for degradation by the 26S proteasome.	Mutant UBB inhibition and decreased function of ubiquitin-proteasome system is a common hallmark of AD. Accumulates in neuronal tau plaques. [15]
	VCP	Valosin Containing Protein	Member of the AAA ATPase family of proteins. plays a role in protein degradation, intracellular membrane fusion, DNA repair and replication, regulation of the cell cycle, and activation of the NF-kappa B pathway.	Involved in ubiquitinated proteins degradation, autophagy, lysosomal clearance and mitochondrial quality control. Mutations in VCP have been linked to ALS and FTD. [16]
**Group 2**				
	ACTB	Actin Beta	Major constituent of the contractile apparatus and one of the two non-muscle cytoskeletal actins that are ubiquitously expressed	--
	ACTR2 (Arp2)	Actin Related Protein 2	ATP-binding component of the Arp2/3 complex, a multiprotein complex that	--

			mediates actin polymerization upon stimulation by nucleation-promoting factor	
	ACTR3	Actin Related Protein 3	ATP-binding component of the Arp2/3 complex, a multiprotein complex that mediates actin polymerization upon stimulation by nucleation-promoting factor	--
	ARPC1A	Actin Related Protein 2/3 Complex Subunit 1A	One of seven subunits of the human Arp2/3 protein complex, involved in regulation of actin polymerization and together with an activating nucleation-promoting factor.	--
	CAPZA1	Capping Actin Protein Of Muscle Z-Line Subunit Alpha 1	Member of the F-actin capping protein alpha subunit family. regulates growth of the actin filament by capping the barbed end of growing actin filaments.	--
	CDC42	Cell Division Cycle 42	Small GTPase of the Rho-subfamily, which regulates signaling pathways that control diverse cellular functions including cell morphology, migration, endocytosis and cell cycle progression. could regulate actin polymerization through its direct binding to Neural Wiskott-Aldrich syndrome protein (N-WASP), which subsequently activates Arp2/3 complex.	Rho GTPase whose specific involvement in AD is debated. Review. [17]
	CFL1	Cofilin 1	Binds to F-actin and exhibits pH-sensitive F-actin depolymerizing activity. Involved in the translocation of actin-cofilin complex from cytoplasm to nucleus	Regulates actin cytoskeleton dynamics, appears to be involved with many steps in neurotoxicity process found in AD. Dysfunction may be related to cytoskeleton stress. [18]
	H2AFX	H2A.X Variant Histone	Replication-independent histone that is a member of the histone H2A family. Variant histone H2A which replaces conventional H2A in a subset of nucleosomes.	--
	H2AFZ	H2A.Z Variant Histone 1	Variant histone H2A which replaces conventional H2A in a subset of nucleosomes.	--
	HIST1H4A (H4C1)	H4 Clustered Histone 1	replication-dependent histone that is a member of the histone H4 family. Core component of nucleosome. Nucleosomes wrap and compact DNA	--

			into chromatin, limiting DNA accessibility to the cellular machineries which require DNA as a template.	
	NAP1L1	Nucleosome Assembly Protein 1 Like 1	Participates in DNA replication and may play a role in modulating chromatin formation and contribute to the regulation of cell proliferation. Histone chaperone that plays a role in the nuclear import of H2A-H2B and nucleosome assembly.	--
	NAP1L4	Nucleosome Assembly Protein 1 Like 4	Member of the nucleosome assembly protein family which can interact with both core and linker histones. histone chaperone in nucleosome assembly.	--
	YWHAB	Tyrosine 3-Monooxygenase/Tryptophan 5-Monooxygenase Activation Protein Beta	to the 14-3-3 family of proteins, members of which mediate signal transduction by binding to phosphoserine-containing proteins. shown to interact with RAF1 and CDC25 phosphatases, suggesting that it may play a role in linking mitogenic signaling and the cell cycle machinery	Identified in protein-protein interacting networks built in OMIM linking AD and BPSD [5]
	YWHAE	Tyrosine 3-Monooxygenase/Tryptophan 5-Monooxygenase Activation Protein Epsilon	Belongs to the 14-3-3 family of proteins which mediate signal transduction by binding to phosphoserine-containing proteins. interacts with CDC25 phosphatases, RAF1 and IRS1 proteins, suggesting its role in diverse biochemical activities related to signal transduction, such as cell division and regulation of insulin sensitivity.	Hub gene in AD identified through differential expression analysis and formation of protein-protein interaction networks. [19]
	YWHAG	Tyrosine 3-Monooxygenase/Tryptophan 5-Monooxygenase Activation Protein Gamma	Belongs to the 14-3-3 family of proteins which mediate signal transduction by binding to phosphoserine-containing proteins. shown to interact with RAF1 and protein kinase C, proteins involved in various signal transduction pathways.	Identified as a candidate biomarker in AD. Observed in higher abundance in AD. [20]
	YWHAH	Tyrosine 3-Monooxygenase/Tryptophan	belongs to the 14-3-3 family of proteins which mediate signal transduction by	Hub gene in AD identified through differential expression analysis and

		5-Monooxygenase Activation Protein Eta	binding to phosphoserine-containing proteins.	formation of protein-protein interaction networks. [19]
	YWHAQ	Tyrosine 3-Monooxygenase/Tryptophan 5-Monooxygenase Activation Protein Theta	belongs to the 14-3-3 family of proteins which mediate signal transduction by binding to phosphoserine-containing proteins.	Hub gene in AD identified through differential expression analysis and formation of protein-protein interaction networks. [19]
	YWHAZ	Tyrosine 3-Monooxygenase/Tryptophan 5-Monooxygenase Activation Protein Zeta	belongs to the 14-3-3 family of proteins which mediate signal transduction by binding to phosphoserine-containing proteins.	Hub gene in AD identified through differential expression analysis and formation of protein-protein interaction networks. [19]
**Group 3**				
	ANXA1	Annexin 1	Membrane-localized protein that binds phospholipids. This protein inhibits phospholipase A2 and has anti-inflammatory activity. Plays important roles in the innate immune response as effector of glucocorticoid-mediated responses and regulator of the inflammatory process.	Inhibits the formation of $A\beta_{1-42}$ via enzymatic degradation by Neprilysin, thereby suppressing the activation of microglial cells in the early stages of the disease. [21]
	ANXA2	Annexin A2	Functions as an autocrine factor which heightens osteoclast formation and bone resorption. A2 expression has been found to correlate with resistance to treatment against various cancer forms	Tau-AnxA2 interaction contributes to enrichment of tau in the axon and is involved in its redistribution in pathological conditions. [22] Potential interacting partner of tau. [23]
	ANXA6	Annexin A6	Calcium-dependent membrane and phospholipid binding protein. Several members of the annexin family have been implicated in membrane-related events along exocytotic and endocytotic pathways. May associate with CD21. May regulate the release of Ca(2+) from intracellular stores.	*See AnxA2* Tau-AnxA2 (AnxA6) interaction contributes to enrichment of tau in the axon and is involved in its redistribution in pathological conditions. [22]
	COL1A2	Collagen Type I Alpha 2 Chain	Pro-alpha2 chain of type I collagen whose triple helix comprises two alpha1 chains and one alpha2 chain. Type I is a fibril-forming collagen found in most connective tissues and is abundant in bone, cornea, dermis and tendon.	--
	COL3A1	Collagen Type III Alpha 1 Chain	Pro-alpha1 chains of type III collagen, a fibrillar collagen that is found in extensible connective tissues such as skin, lung, uterus, intestine and the	--

			vascular system, frequently in association with type I collagen.	
	COL4A1	Collagen Type IV Alpha 1 Chain	A type IV collagen alpha protein, integral component of basement membranes. Part of a heterotrimer and interacts with other extracellular matrix components such as perlecans, proteoglycans, and laminins. Proteolytic cleavage of the non-collagenous carboxy-terminal domain results in a biologically active fragment known as arresten, which has anti-angiogenic and tumor suppressor properties.	--
	COL4A2	Collagen Type IV Alpha 2 Chain	One of six subunits of type IV collagen, structural component of basement membranes. C-terminal portion of the protein, known as canstatin, is an inhibitor of angiogenesis and tumor growth.	--
	GNA11	G Protein Subunit Alpha 11	alpha-11 subunit of G protein. Modulation and transduction in various transmembrane signaling systems.	--
	GNA13	G Protein Subunit Alpha 13	Predicted to enable D5 dopamine receptor binding activity; G-protein beta/gamma-subunit complex binding activity; and GTPase activity.	Identified as a key biomarker for a gene module associated with accumulation of Abeta and p-tau. [24]
	GNAI2	G Protein Subunit Alpha I2	Contains the guanine nucleotide binding site and is involved in the hormonal regulation of adenylate cyclase.	--
	GNAI3	G Protein Subunit Alpha I3	Alpha subunit and belongs to the G-alpha family. Mutation in this gene, resulting in a gly40-to-arg substitution, is associated with auriculocondylar syndrome, and shown to affect downstream targets in the G protein-coupled endothelin receptor pathway.	Identified in protein subsets in AD and AsymAD vs control using machine learning. Tandon, 2023 [25]
	GNAO1	G Protein Subunit Alpha O1	Alpha subunit of the Go heterotrimeric G-protein signal-transducing complex. Defects in this gene are a cause of early-onset epileptic encephalopathy.	--
	GNAS	GNAS Complex Locus	May inhibit the adenylyl cyclase-stimulating activity of guanine	--

			nucleotide-binding protein G(s) subunit alpha which is produced from the same locus in a different open reading frame.	
	GNB1	G Protein Subunit Beta 1	G protein beta subunits are important regulators of alpha subunits, as well as of certain signal transduction receptors and effectors	Identified as a hub gene that might be major regulator for TGFBR3 functions in AD. [26]
	GNB2	G Protein Subunit Beta 2	Beta chains are required for the GTPase activity, for replacement of GDP by GTP, and for G protein-effector interaction	--
	GNG12	G Protein Subunit Gamma 12	Enables PDZ domain binding activity. Predicted to be involved in G protein-coupled receptor signaling pathway. Located in extracellular exosome.	--
	GNG2	G Protein Subunit Gamma 2	related pathways are ADORA2B mediated anti-inflammatory cytokines production and Thromboxane signalling through TP receptor. Gene Ontology (GO) annotations related to this gene include *obsolete signal transducer activity* and *G-protein beta-subunit binding*.	--
	MMP2	Matrix Metallopeptidase 2	Proteins in this family are involved in the breakdown of extracellular matrix in normal physiological processes, such as embryonic development, reproduction, and tissue remodeling, as well as in disease processes, such as arthritis and metastasis.	MMP-2 directly degrades Aβ resulting in the clearance of Aβ deposits, MMP-2 induces breakdown of BBB, and this deleterious effect could be reversed by TIMP-2, MMP-2 has both proinflammatory /pro-angiogenetic and anti-inflammatory/ anti-angiogenetic effects on AD. [27] Overexpression observed in postmortem brain from AD patients. [28]

References:

1. Beckelman BC, Zhou X, Keene CD, Ma T. Impaired Eukaryotic Elongation Factor 1A Expression in Alzheimer's Disease. *Neurodegener Dis* 2016;16:39–43. [PMID: 26551858 DOI: 10.1159/000438925].

2. Shigemizu D, Mori T, Akiyama S, *et al.* Identification of potential blood biomarkers for early diagnosis of Alzheimer's disease through RNA sequencing analysis. *Alzheimers Res Ther* 2020;12:87. [PMID: 32677993 DOI: 10.1186/s13195-020-00654-x].

3. Li X, Alafuzoff I, Soininen H, Winblad B, Pei J-J. Levels of mTOR and its downstream targets 4E-BP1, eEF2, and eEF2 kinase in relationships with tau in Alzheimer's disease brain. *FEBS J* 2005;272:4211–20. [PMID: 16098202 DOI: 10.1111/j.1742-4658.2005.04833.x].

4. Lopez-Toledo G, Silva-Lucero M-D-C, Herrera-Díaz J, García D-E, Arias-Montaño J-A, Cardenas-Aguayo M-D-C. Patient-Derived Fibroblasts With Presenilin-1 Mutations, That Model Aspects of Alzheimer's Disease Pathology, Constitute a Potential Object for Early Diagnosis. *Front Aging Neurosci* 2022;14:921573. [PMID: 35847683 DOI: 10.3389/fnagi.2022.921573].

5. Mao Y, Fisher DW, Yang S, Keszycki RM, Dong H. Protein-protein interactions underlying the behavioral and psychological symptoms of dementia (BPSD) and Alzheimer's disease. *PLoS One* 2020;15:e0226021. [PMID: 31951614 DOI: 10.1371/journal.pone.0226021].

6. Gonzalez-Rodriguez M, Villar-Conde S, Astillero-Lopez V, *et al.* Neurodegeneration and Astrogliosis in the Human CA1 Hippocampal Subfield Are Related to hsp90ab1 and bag3 in Alzheimer's Disease. *Int J Mol Sci* 2021;23:165. [PMID: 35008592 DOI:10.3390/ijms23010165].

7. Lalwani AK, Krishnan K, Bagabir SA, *et al.* Network Theoretical Approach to Explore Factors Affecting Signal Propagation and Stability in Dementia's Protein-Protein Interaction Network. *Biomolecules* 2022;12:451. [PMID: 35327643 DOI: 10.3390/biom12030451].

8. Moreno JA, Tiffany-Castiglioni E. The chaperone Grp78 in protein folding disorders of the nervous system. *Neurochem Res* 2015;40:329–35. [PMID: 25107299 DOI: 10.1007/s11064-014-1405-0].

9. Kumar P, Jha NK, Jha SK, Ramani K, Ambasta RK. Tau phosphorylation, molecular chaperones, and ubiquitin E3 ligase: clinical relevance in Alzheimer's disease. *J Alzheimers Dis* 2015;43:341–61. [PMID: 25096626 DOI: 10.3233/JAD-140933].

10. Li J, Zhang Y, Lu T, *et al.* Identification of diagnostic genes for both Alzheimer's disease and Metabolic syndrome by the machine learning algorithm. *Front Immunol* 2022;13:1037318. [PMID: 36405716 DOI: 10.3389/fimmu.2022.1037318].

11. Tao Y, Han Y, Yu L, Wang Q, Leng SX, Zhang H. The Predicted Key Molecules, Functions, and Pathways That Bridge Mild Cognitive Impairment (MCI) and Alzheimer's Disease (AD). *Front Neurol* 2020;11:233. [PMID: 32308643 DOI: 10.3389/fneur.2020.00233].

12. Wu Y, Zhang S, Xu Q, *et al.* Regulation of global gene expression and cell proliferation by APP. *Sci Rep* 2016;6:22460. [PMID: 26936520 DOI: 10.1038/srep22460].

13. Ji W, An K, Wang C, Wang S. Bioinformatics analysis of diagnostic biomarkers for Alzheimer's disease in peripheral blood based on sex differences and support vector machine algorithm. *Hereditas* 2022;159:38. [PMID: 36195955 DOI: 10.1186/s41065-022-00252-x].

14. Xu H, Jia J. Immune-Related Hub Genes and the Competitive Endogenous RNA Network in Alzheimer's Disease. *J Alzheimers Dis* 2020;77:1255–65. [PMID: 32925027 DOI: 10.3233/JAD-200081].

15. Chojnacki M, Zhang D, Talarowska M, *et al.* Characterizing polyubiquitinated forms of the neurodegenerative ubiquitin mutant UBB+1. *FEBS Lett* 2016;590:4573–85. [PMID: 27861798 DOI: 10.1002/1873-3468.12484].

16. Scarian E, Fiamingo G, Diamanti L, Palmieri I, Gagliardi S, Pansarasa O. The Role of VCP Mutations in the Spectrum of Amyotrophic Lateral Sclerosis-Frontotemporal Dementia. *Front Neurol* 2022;13:841394. [PMID: 35273561 DOI: 10.3389/fneur.2022.841394].

17. Aguilar BJ, Zhu Y, Lu Q. Rho GTPases as therapeutic targets in Alzheimer's disease. *Alzheimers Res Ther* 2017;9:97. [PMID:29246246 DOI: 10.1186/s13195-017-0320-4].

18. Schönhofen P, de Medeiros LM, Chatain CP, Bristot IJ, Klamt F. Cofilin/actin rod formation by dysregulation of cofilin-1 activity as a central initial step in neurodegeneration. *Mini Rev Med Chem* 2014;14:393–400. [PMID: 24813767 DOI: 10.2174/1389557514666140506161458].

19. Yang F, Diao X, Wang F, *et al.* Identification of Key Regulatory Genes and Pathways in Prefrontal Cortex of Alzheimer's Disease. *Interdiscip Sci* 2020;12:90–8. [PMID: 32006383 DOI: 10.1007/s12539-019-00353-8].

20. Sathe G, Na CH, Renuse S, *et al.* Quantitative Proteomic Profiling of Cerebrospinal Fluid to Identify Candidate Biomarkers for Alzheimer's Disease. *Proteomics Clin Appl* 2019;13:e1800105. [PMID: 30578620 DOI: 10.1002/prca.201800105].

21. Jayaswamy PK, Vijaykrishnaraj M, Patil P, Alexander LM, Kellarai A, Shetty P. Implicative role of epidermal growth factor receptor and its associated signaling partners in the pathogenesis of Alzheimer's disease. *Ageing Res Rev* 2023;83:101791. [PMID: 36403890 DOI: 10.1016/j.arr.2022.101791].

22. Gauthier-Kemper A, Suárez Alonso M, Sündermann F, *et al.* Annexins A2 and A6 interact with the extreme N terminus of tau and thereby contribute to tau's axonal localization. *J Biol Chem* 2018;293:8065–76. [PMID: 29636414 DOI:10.1074/jbc.RA117.000490].

23. Gauthier-Kemper A, Weissmann C, Golovyashkina N, *et al.* The frontotemporal dementia mutation R406W blocks tau's interaction with the membrane in an annexin A2-dependent manner. *J Cell Biol* 2011;192:647–61. [PMID: 21339331 DOI:10.1083/jcb.201007161].

24. Zhang T, Shen Y, Guo Y, Yao J. Identification of key transcriptome biomarkers based on a vital gene module associated with pathological changes in Alzheimer's disease. *Aging (Albany NY)* 2021;13:14940–67. [PMID: 34031265 DOI: 10.18632/aging.203017].

25. Tandon R, Levey AI, Lah JJ, Seyfried NT, Mitchell CS. Machine Learning Selection of Most Predictive Brain Proteins Suggests Role of Sugar Metabolism in Alzheimer's Disease. *J Alzheimers Dis* 2023. [PMID: 36776048 DOI: 10.3233/JAD-220683].

26. Song H, Yang J, Yu W. Promoter Hypomethylation of TGFBR3 as a Risk Factor of Alzheimer's Disease: An Integrated Epigenomic-Transcriptomic Analysis. *Front Cell Dev Biol* 2021;9:825729. [PMID: 35310542 DOI: 10.3389/fcell.2021.825729].

27. Wang H, Huang L, Wu L, *et al.* The MMP-2/TIMP-2 System in Alzheimer Disease. *CNS Neurol Disord Drug Targets* 2020;19:402–16. [PMID: 32787764 DOI: 10.2174/1871527319666200812223007].

28. Rempe RG, Hartz AMS, Bauer B. Matrix metalloproteinases in the brain and blood-brain barrier: Versatile breakers and makers. *J Cereb Blood Flow Metab* 2016;36:1481–507. [PMID: 27323783 DOI: 10.1177/0271678X16655551].

Supplementary Table 3. Functions of proteins present at similar levels in mTau and mPS1 EVs

Proteins found in both mTau and mPS1 exosomes and present at similar levels in these exosomes are shown in this Table for their gene names, protein names, functions, and examples of their association in Alzheimer's disease (AD) or tauopathies.

Gene name	Protein name	Normal functions	Functions related to AD or tauopathies
ALDH2	Aldehyde dehydrogenase	Alcohol metabolism	ALDH2 participates in oxidative stress and memory deficits shown by mouse knockout of ALDH2 [1].
ANXA2	Annexin A2	Cell growth	Annexins A2 and A6 interact with the extreme N terminus of tau and thereby contribute to tau's axonal localization [2].
CLU	Clusterin	Cell death	CLU is a genetic modifier for late-onset Alzheimer's disease [3].
EEF1A1	Elongation factor 1-alpha	RNA transport	EEF1A1 expression is reduced in AD patients in the hippocampus [4].
FSCN1	Fascin actin-bundling protein 1	Cell migration	FSCN1 shows altered expression in brains with Alzheimer's disease [5].
GDI1	GDP dissociation inhibitor 1	Vesicular trafficking	Mutated PS-1 impacts membrane transport by binding to rabGDI and dysregulates production of Abeta [6].
HSPA8	Heat shock protein family A member 8	Protein folding	HSPA8 is significantly downregulated in Alzheimer's disease brains and is a potential molecular biomarker for prognosis [7].
IIH4	General transcription factor IIH subunit 4	DNA repair	
LGALS3BP	Galectin 3 binding protein	Modulating cell-cell interactions	Galectin 3-binding protein suppresses amyloid-ß production by modulating ß-cleavage of amyloid precursor protein [8].
MARCKS	Myristolylated alanine rich protein kinase C substrate	Actin filament linkage	MARCKS phosphorylation is decreased in AD brains but increased in microglia and amyloid beta-protein deposits [9].
PARK7	Parkinsonism associated deglycase	Transcription regulation	DJ-1, which is encoded in PARK7, has been shown to have therapeutic effects on neurodegenerative disorders such as AD [10].
TUBB4A	Tubulin beta-4A chain	Microtubule formation	TUBB4A was found to be correlated with tau inclusion formation, specifically tau isolated from AD brains [11].
VCL	Vinculin	Actin filament anchorage	VCL is upregulated in individuals with Alzheimer's neuropathology compared to control [12].
YWHAE	Tyrosine 3-monooxygenase/tryptophan 5-	Signal transduction	YWHAE may be able to explain how neurotropism triggers neurodegeneration and

	monooxygenase activation protein epsilon		leads to neurodegenerative diseases such as AD [13].
YWHAH	Tyrosine 3-monooxygenase/tryptophan 5-monooxygenase activation	Signal transduction	YWHAH is consistently downregulated in the three groups of differentially expressed genes [14].

References

1. D'Souza Y, Elharram A, Soon-Shiong R, Andrew RD, Bennett BM. Characterization of Aldh2 (-/-) mice as an age-related model of cognitive impairment and Alzheimer's disease. *Mol Brain* 2015;8:27. [PMID: 25910195 DOI: 10.1186/s13041-015-0117-y].

2. Gauthier-Kemper A, Suárez Alonso M, Sündermann F, *et al.* Annexins A2 and A6 interact with the extreme N terminus of tau and thereby contribute to tau's axonal localization. *J Biol Chem* 2018;293:8065–76. [PMID: 29636414 DOI: 10.1074/jbc.RA117.000490].

3. Chen F, Swartzlander DB, Ghosh A, Fryer JD, Wang B, Zheng H. Clusterin secreted from astrocyte promotes excitatory synaptic transmission and ameliorates Alzheimer's disease neuropathology. *Mol Neurodegener* 2021;16:5. [PMID: 33517893 DOI: 10.1186/s13024-021-00426-7].

4. Beckelman BC, Zhou X, Keene CD, Ma T. Impaired Eukaryotic Elongation Factor 1A Expression in Alzheimer's Disease. *Neurodegener Dis* 2016;16:39–43. [PMID: 26551858 DOI: 10.1159/000438925].

5. Kloske CM, Dugan AJ, Weekman EM, *et al.* Inflammatory Pathways Are Impaired in Alzheimer Disease and Differentially Associated With Apolipoprotein E Status. *J Neuropathol Exp Neurol* 2021;80:922–32. [PMID: 34486652 DOI: 10.1093/jnen/nlab085].

6. Scheper W, Zwart R, van der Sluijs P, Annaert W, Gool WA, Baas F. Alzheimer's presenilin 1 is a putative membrane receptor for rab GDP dissociation inhibitor. *Hum Mol Genet* 2000;9:303–10. [PMID: 10607841 DOI: 10.1093/hmg/9.2.303].

7. Dong Y, Li T, Ma Z, Zhou C, Wang X, Li J. HSPA1A, HSPA2, and HSPA8 Are Potential Molecular Biomarkers for Prognosis among HSP70 Family in Alzheimer's Disease. *Dis Markers* 2022;2022:9480398. [PMID: 36246562 DOI: 10.1155/2022/9480398].

8. Seki T, Kanagawa M, Kobayashi K, *et al.* Galectin 3-binding protein suppresses amyloid-β production by modulating β-cleavage of amyloid precursor protein. *J Biol Chem* 2020;295:3678–91. [PMID: 31996371 DOI: 10.1074/jbc.RA119.008703].

9. Kimura T, Yamamoto H, Takamatsu J, Yuzuriha T, Miyamoto E, Miyakawa T. Phosphorylation of MARCKS in Alzheimer disease brains. *Neuroreport* 2000;11:869–73. [PMID: 10757536 DOI: 10.1097/00001756-200003200-00042].

10. Hijioka M, Inden M, Yanagisawa D, Kitamura Y. DJ-1/PARK7: A New Therapeutic Target for Neurodegenerative Disorders. *Biol Pharm Bull* 2017;40:548–52. [PMID: 28458339 DOI: 10.1248/bpb.b16-01006].

11. Ficulle E, Kananathan S, Airey D, *et al.* A human tau seeded neuronal cell model recapitulates molecular responses associated with Alzheimer's disease. *Sci Rep* 2022;12:2673. [PMID: 35177665 DOI: 10.1038/s41598-022-06411-4].

12. Zolochevska O, Bjorklund N, Woltjer R, Wiktorowicz JE, Taglialatela G. Postsynaptic Proteome of Non-Demented Individuals with Alzheimer's Disease Neuropathology. *J Alzheimers Dis* 2018;65:659–82. [PMID: 30103319 DOI: 10.3233/JAD-180179].

13. Vavougios GD. SARS-CoV-2 dysregulation of PTBP1 and YWHAE/Z gene expression: A primer of neurodegeneration. *Med Hypotheses* 2020;144:110212. [PMID: 33254518 DOI: 10.1016/j.mehy.2020.110212].

14. Williams JB, Cao Q, Yan Z. Transcriptomic analysis of human brains with Alzheimer's disease reveals the altered expression of synaptic genes linked to cognitive deficits. *Brain Commun* 2021;3:fcab123. [PMID: 34423299 DOI: 10.1093/braincomms/fcab123].

Supplementary Table 4. Downregulated and upregulated proteins in mTau compared to mPS1 EVs

Exosome proteins that are downregulated or upregulated in mTau compared to mPS1 exosomes (shown in Figure 3) are provided in this Table for information of gene ad protein names, normal functions, and functions related to AD or tauopathies.

Gene name	Protein name	Normal functions	Functions related to AD or tauopathies
**Downregulated in mTau compared to mPS1 exosomes:**			
GSN	Gelsolin	Binds to the “plus” ends of actin monomers and filaments to prevent monomer exchange	Forms complex with amyloid-beta protein and reduces amyloid load in transgenic mouse AD model [1]. Gelsolin binds Aβ, inhibits its aggregation into fibrils, and protects cells from apoptosis induced by Aβ. In context of AD, it cleaved by caspase-3 during apoptosis. Appearance of gelsolin-carboxy-terminal fragments found in frontal cortex positively correlated with severity of AD [1, 2].
ATP1A1	ATPase Na+/K+ Transporting Subunit Alpha 1	Establishes and maintains electrochemical gradients of Na and K ions across the plasma membrane (this gene encodes an alpha 1 subunit)	Na+-K+-ATPase is a potent neuroprotective modulator against AD, responsible for synaptic plasticity such as long-term potentiation [3]. Factors such as β-amyloid, cholinergic, and oxidative stress can modulate learning and memory in AD through reduction of Na+-K+-ATPase activity [4].
PSMA6	Proteasome 20S Subunit Alpha 6	Multicatalytic proteinase complex with a highly ordered ring-shaped 20S core structure. Cleaves peptides in an ATP/ubiquitin-dependent process in a non-lysosomal pathway	Amyloid accumulation directly inhibits proteasome activity [5]. Tau also blocks proteolysis by the 20S core Proteasome, demonstrating that the Proteasome interacts with Tau during the disease state and accumulation of tau further inhibits the Proteasome [6].
OLFM1	Olfactomedin 1	Glycoprotein preferentially expressed in neuronal tissue. Exact function is unknown, but hypotheses include inhibition of interactions between RTN4R and LINGO1 and regulation of production of neural crest cells by the neural tube	Olfm1 may interact with amyloid precursor protein (APP), suppress its cleavage, and inhibit the subsequent production of Aβ through interaction with BACE1 [7].<br><br>Olfactomedin 1 interacts with amyloid precursor protein and modulates cortical cell migration during development [7]. Deletion of N-terminal half of Olfactomedin 1 modifies interaction with synaptic proteins and causes brain atrophy. Modified interactions of Olfm1 with binding targets leads to increase in Ca2+ concentration and activation of ERK1/2, MEK1, and CaMKII in hippocampus of *Olfm1* mutant mice compared to wild-type mice [8]

PLOD1	Procollagen-Lysine, 2-Oxoglutaratre 5-Dioxygenase 1	Membrane-bound homodimeric protein localized to the cisternae of the endoplasmic reticulum. This enzyme (cofactors iron and ascorbate) catalyzes the hydroxylation of lysyl residues in collagen-like peptides	Statistically significant upregulation in PLOD1 for fast compared with slow-progressors of AD subjects across time points [9].
IGHG2	Immunoglobulin Heavy Constant Gamma 2	Encodes the constant (C) region of the gamma-2 heavy chain, which defines the IgG2 isotype	Significantly higher levels of IgM and IgG in late-stage AD (Braak stages V and VI) compared to age-matched controls have been reported. Levels of IgG2 and IgG4 constant region fragments were higher in late-stage AD, with concentrations of native-state IgG4 with free Fc regions increased in Braak stages III and VI [10].<br>In mice studies, however, APOE4-transgenic mice with Aβ accumulation display decreased immunoglobulin G in neocortex, entorhinal cortex, and hippocampus [11]. This might result from defects of humoral immunity and lead to impairments of IgG-mediated clearance of Aβ by microglia, consequently facilitating AD progression.
MATN2	Matrillin 2	Member of the von Willebrand factor A domain containing protein family. Believed to be involved in formation of filamentous networks in the extracellular matrices of various tissues	--
A2M	Alpha-2-Macroglobulin	Protease inhibitor and cytokine transporter	This gene is implicated in AD due to its ability to mediate the clearance and degradation of Aβ. α-2M binds tightly to Aβ peptide and attenuates fibrillogenesis and neurotoxicity of Aβ [12, 13].
GPC2	Glypican 2	Predicted to be involved in several processes, including positive regulation of neuron projection development, regulation of protein localization to membrane, and smoothened signaling pathways. Located in endoplasmic reticulum	Empirical evidence suggests that glypican receptor mediates beta-amyloid neurotoxicity in PC12 cells by binding Aβ on the neuronal cell membrane and as a potential source of heparan sulfate found in AD neurofibrillary tangles and senile plaques [14].
CFL1	Cofilin 1	Polymerizes and depolymerizes F-actin and G-actin in a pH-dependent manner.	Cofilin in Hirano bodies and cofilin-actin rods increases with age and AD and animal models of AD [15]. Cofilin-1 phosphorylation (inactivation) by Aβ-mediated activation of LIMK1 causes loss of dendritic spine in hippocampus [16].
CRMP1	Collasin Response Mediator Protein 1	Family of dihydropyrimidinase-related neuronal proteins involved in axonal outgrowth	Overaction of NMDA receptors and subsequent calcium influx and of Cdk5 and GSK3B hyper phosphorylates CRMP2 (which shares 75% homology with CRMP1) and leads to dysregulated microtubule dynamics and reduced neurite elongation [17, 18].
ALB	Albumin	Most abundant protein in human blood. Functions in regulation of blood colloid osmotic pressure and acts as a carrier protein	Low serum albumin is associated with increased odds of cognitive impairment in the elderly [19].
YWHAG	Tyrosine 3-Monooxygenase/Try	Mediates signal transduction by binding to phosphoserine-containing proteins	For high-resolution mass spectrometry and tandem mass tag (TMT) evaluation of novel biomarkers for AD,

	ptophan 5-Monooxygenase Activation Protein Gamma		YWHAG, Gelsolin (GSN), and pyruvate kinase (PKM) showed significant increases. YWHAG showed 2.2-fold higher levels in AD CSF samples than controls ($p<0.0001$) [20].
DIP2B	Disco Interacting Protein 2 Homolog B	Protein that contains a binding site for the transcriptional regulator DNA methyltransferase 1 associated protein 1 as well as AMP-binding site (DNA methylation processes)	Global and gene-specific DNA methylation patterns are altered in various brain regions of individuals with AD [21]. Higher Aβ plaque burden associated with promoter hypomethylation of the Presenilin enhancer 2 (PEN-2) gene, one of the rate-limiting genes in the formation of gamma-secretase [22].
PKM	Pyruvate kinase M1/2	Glycolysis protein. Catalyzes the transfer of a phosphoryl group from phosphoenolpyruvate to ADP, generating ATP and pyruvate	For high-resolution mass spectrometry and tandem mass tag (TMT) evaluation of novel biomarkers for AD, YWHAG, Gelsolin (GSN), and pyruvate kinase (PKM) showed significant increases [20]. Pyruvate kinase M2 is a positive regulator of gamma-secretase under hypoxia and regulates AB production [23].
**Upregulated in mTau compared to mPS1 exosomes:**			
HSPG2	Heparan Sulfate Proteoglycan 2 (Perlecan)	Core protein to which three long chains of glycosaminoglycans are attached. It is a large multidomain proteoglycan that binds to and cross-links many extracellular matrix components	Under physiological conditions tau and GAGs would never meet, however recent work shows tau aggregates can spread like prions in the brain and be taken up by neurons and propagated. This uptake is mediated by heparan sulfate [24].
COL4A2	Collagen Type IV Alpha 2 Chain	One of the six subunits of type IV collagen, the major structural component of basement membranes	--
ACTB	Actin Beta	Involved in cell motility, structure, integrity, and intercellular signaling. This protein is a major constituent of the contractile apparatus and one of the two non-muscle cytoskeletal actins that are ubiquitously expressed	Activation of Rho-associated protein kinase (ROCK) pathway results in phosphorylation of cof1 (neuronal actin-binding protein) and is sufficient to mediate Aβ-induced aberrant F-actin depolarization, leading to synaptic impairment and synaptic loss within dendritic spines [25].
IGHA1	Immunoglobulin Heavy Constant Alpha 1	Encodes a constant (C) segment of Immunoglobulin A heavy chain. Immunoglobulin A is an antibody that plays a critical role in immune function in the mucous membranes	Plasma IgA levels higher in AD patients compared to normal controls (NC). Also, AD patients demonstrated higher IgA area fraction and IgA+ cell number compared to NC. When *APOE4* status considered, higher plasma IgA levels in AD patients were only seen in *APOE4* non-carriers. Plasma IgA levels in *APOE4* non-carriers associated with cognitive decline [26].
SPON1	Spondin 1	Extracellular matrix structural constituent. Predicted to be involved in cell adhesion	Spondin 1 can reduce amyloid beta and reverse cognitive impairment and memory dysfunction in vitro in neural cells and in in-vivo models [27]. Spondin-1 has been shown to bind to the extracellular domain of AβPP, inhibiting its cleavage by beta-secretase [28].

TF	Transferrin	Glycoprotein with C and N-terminal domains each of which bind one ion of ferric iron. Transports iron from intestine, reticuloendothelial system, and liver parenchymal cells to all proliferating cells in the body	In a longitudinal analysis, higher plasma transferrin was associated with a steeper cognitive decline in the mild cognitive impairment (MCI) and AD groups, but not in the normal cognition group [29].

References

1. Ji L, Chauhan A, Wegiel J, Essa MM, Chauhan V. Gelsolin is proteolytically cleaved in the brains of individuals with Alzheimer's disease. *J Alzheimers Dis.* 2009;18(1):105-11. [PMID: 19625752 DOI: 10.3233/JAD-2009-1127.]

2. Ji L, Zhao X, Hua Z. Potential therapeutic implications of gelsolin in Alzheimer's disease. *J Alzheimers Dis*. 2015;44(1):13-25. [PMID: 25208622 DOI: 10.3233/JAD-141548.]

3. Zhang LN, Sun YJ, Pan S, et al. Na^+-K^+-ATPase, a potent neuroprotective modulator against Alzheimer disease. *Fundam Clin Pharmacol*. 2013 Feb;27(1):96-103. [PMID: 23033963 DOI: 10.1111/fcp.12000.]

4. Moseley AE, Williams MT, Schaefer TL, et al. Deficiency in Na,K-ATPase alpha isoform genes alters spatial learning, motor activity, and anxiety in mice. *J Neurosci*. 2007 Jan 17;27(3):616-26. [PMID: 17234593 DOI: 10.1523/JNEUROSCI.4464-06.2007.]

5. Tseng BP, Green KN, Chan JL, Blurton-Jones M, LaFerla FM. Abeta inhibits the proteasome and enhances amyloid and tau accumulation. *Neurobiol Aging*. 2008 Nov;29(11):1607-18.[PMID: 17544172 DOI: 10.1016/j.neurobiolaging.2007.04.014.]

6. Keck S, Nitsch R, Grune T, Ullrich O. Proteasome inhibition by paired helical filament-tau in brains of patients with Alzheimer's disease. *J Neurochem*. 2003 Apr;85(1):115-22. [PMID: 12641733 DOI: 10.1046/j.1471-4159.2003.01642.x.]

7. Rice HC, Townsend M, Bai J, Suth S, Cavanaugh W, Selkoe DJ, et al. Pancortins interact with amyloid precursor protein and modulate cortical cell migration. Development. 2012 Nov;139(21):3986-96. [PMID: 22992957 DOI: 10.1242/dev.082909.]

8. Nakaya N, Sultana A, Munasinghe J, Cheng A, Mattson MP, Tomarev SI. Deletion in the N-terminal half of olfactomedin 1 modifies its interaction with synaptic proteins and causes brain dystrophy and abnormal behavior in mice. *Exp Neurol*. 2013 Dec; 250:205-18. [PMID: 24095980 DOI: 10.1016/j.expneurol.2013.09.019.]

9. Chong MS, Goh LK, Lim WS, et al. Gene expression profiling of peripheral blood leukocytes shows consistent longitudinal downregulation of TOMM40 and upregulation of KIR2DL5A, PLOD1, and SLC2A8 among fast progressors in early Alzheimer's disease. *J Alzheimers Dis*. 2013;34(2):399-405. [PMID: 23234877 DOI: 10.3233/JAD-121621.]

10. Lekhraj R, Lalezari S, Aguilan JT, et al. Altered abundances of human immunoglobulin M and immunoglobulin G subclasses in Alzheimer's disease frontal cortex. *Sci Rep*. 2022 Apr 28;12(1):6934. [PMID: 35484384 DOI: 10.1038/s41598-022-10793-w.]

11. Zhang L, Xu J, Gao J, Chen P, Yin M, Zhao W. Decreased immunoglobulin G in brain regions of elder female APOE4-TR mice accompany with Aβ accumulation. *Immun Ageing*. 2019 Jan 25;16:2. [PMID: 30700991 DOI: 10.1186/s12979-018-0142-7.]

12. Du Y, Bales KR, Dodel RC, et al. Alpha2-macroglobulin attenuates beta-amyloid peptide 1-40 fibril formation and associated neurotoxicity of cultured fetal rat cortical neurons. *J Neurochem*. 1998 Mar;70(3):1182-8. [PMID: 9489740. DOI: 10.1046/j.1471-4159.1998.70031182.x.]

13. Hughes SR, Khorkova O, Goyal S, et al. Alpha2-macroglobulin associates with beta-amyloid peptide and prevents fibril formation. *Proc Natl Acad Sci U S A.* 1998 Mar 17;95(6):3275-80. [PMID: 9501253 DOI: 10.1073/pnas.95.6.3275.]

14. Schulz JG, Megow D, Reszka R, Villringer A, Einhäupl KM, Dirnagl U. Evidence that glypican is a receptor mediating beta-amyloid neurotoxicity in PC12 cells. Eur *J Neurosci*. 1998 Jun;10(6):2085-93. [PMID: 9753095 DOI: 10.1046/j.1460-9568.1998.00220.x.]

15. Galloway PG, Perry G, Gambetti P. Hirano body filaments contain actin and actin-associated proteins. *J Neuropathol Exp Neurol*. 1987 Mar;46(2):185-99. [PMID: 3029338 DOI: 10.1097/00005072-198703000-00006.]

16. Heredia L, Helguera P, de Olmos S, et al. Phosphorylation of actin-depolymerizing factor/cofilin by LIM-kinase mediates amyloid beta-induced degeneration: a potential mechanism of neuronal dystrophy in Alzheimer's disease. *J Neurosci*. 2006 Jun 14;26(24):6533-42. [PMID: 16775141 DOI: 10.1523/JNEUROSCI.5567-05.2006.]

17. Arimura N, Ménager C, Kawano Y, et al. Phosphorylation by Rho kinase regulates CRMP-2 activity in growth cones. *Mol Cell Biol*. 2005 Nov;25(22):9973-84. [PMID: 16260611 DOI: 10.1128/MCB.25.22.9973-9984.2005.]

18. Cole AR, Knebel A, Morrice NA, et al. GSK-3 phosphorylation of the Alzheimer epitope within collapsin response mediator proteins regulates axon elongation in primary neurons. J Biol Chem. 2004 Nov 26;279(48):50176-80. PMID: 15466863 DOI: 10.1074/jbc.C400412200.]

19. Menendez-Gonzalez M, Gasparovic C. Albumin Exchange in Alzheimer's Disease: Might CSF Be an Alternative Route to Plasma? *Front Neurol*. 2019 Oct 18;10:1036. [PMID: 31681137 DOI: 10.3389/fneur.2019.01036.]

20. Sathe G, Na CH, Renuse S, et al. Quantitative Proteomic Profiling of Cerebrospinal Fluid to Identify Candidate Biomarkers for Alzheimer's Disease. *Proteomics Clin Appl*. 2019 Jul;13(4):e1800105. [PMID: 30578620 DOI: 10.1002/prca.201800105.]

21. Yokoyama AS, Rutledge JC, Medici V. DNA methylation alterations in Alzheimer's disease. *Environ Epigenet*. 2017 Jun 6;3(2):dvx008. [PMID: 29492310 DOI: 10.1093/eep/dvx008.]

22. Lang AL, Eulalio T, Fox E, et al. Methylation differences in Alzheimer's disease neuropathologic change in the aged human brain. *Acta Neuropathol Commun*. 2022 Nov 29;10(1):174. [PMID: 36447297 DOI: 10.1186/s40478-022-01470-0.]

23. Han J, Hyun J, Park J, Jung S, Oh Y, Kim Y, Ryu SH, Kim SH, Jeong EI, Jo DG, Park SH, Jung YK. Aberrant role of pyruvate kinase M2 in the regulation of gamma-secretase and memory deficits in Alzheimer's disease. *Cell Rep*. 2021 Dec 7;37(10):110102. [PMID: 34879266 DOI: 10.1016/j.celrep.2021.110102.]

24. Holmes BB, DeVos SL, Kfoury N, et al. Heparan sulfate proteoglycans mediate internalization and propagation of specific proteopathic seeds. *Proc Natl Acad Sci U S A*. 2013 Aug 13;110(33):E3138-47. [PMID: 23898162 DOI: 10.1073/pnas.1301440110.]

25. Rush T, Martinez-Hernandez J, Dollmeyer M, et al. Synaptotoxicity in Alzheimer's Disease Involved a Dysregulation of Actin Cytoskeleton Dynamics through Cofilin 1 Phosphorylation. J Neurosci. 2018 Nov 28;38(48):10349-10361. [PMID: 30341179 DOI: 10.1523/JNEUROSCI.1409-18.2018.]

26. Pocevičiūtė D, Nuñez-Diaz C, Roth B, et al. Increased plasma and brain immunoglobulin A in Alzheimer's disease is lost in apolipoprotein E ε4 carriers. Alzheimers Res Ther. 2022 Aug 26;14(1):117. [PMID: 36008818 DOI: 10.1186/s13195-022-01062-z.]

27. Park SY, Kang JY, Lee T, Nam D, Jeon CJ, Kim JB. SPON1 Can Reduce Amyloid Beta and Reverse Cognitive Impairment and Memory Dysfunction in Alzheimer's Disease Mouse Model. Cells. 2020 May 21;9(5):1275. [PMID: 32455709 DOI:10.3390/cells9051275.]

28. Ho A, Südhof TC. Binding of F-spondin to amyloid-beta precursor protein: a candidate amyloid-beta precursor protein ligand that modulates amyloid-beta precursor protein cleavage. Proc Natl Acad Sci U S A. 2004 Feb 24;101(8):2548-53. [PMID: 14983046 DOI: 10.1073/pnas.0308655100.]

29. Guan J, Wang P, Lu L, Zhao G. Association of Plasma Transferrin With Cognitive Decline in Patients With Mild Cognitive Impairment and Alzheimer's Disease. *Front Aging Neurosci*. 2020 Mar 12;12:38. [PMID: 32226377 DOI: 10.3389/fnagi.2020.00038.]

Supplementary Table 5. Hub proteins present only in mPS1 exosomes, and not in mTau EVs.

Protein networks of proteins present in only mPS1 exosomes (not in mTau exosomes) are illustrated in Figure 5. The components of three main hubs are provided in this Table, showing gene and protein names, functions, and functions related to AD or tauopathies.

Hub Group	Gene name	Protein name	Normal functions	Functions related to AD or tauopathies
**Group 1**	CCT2	T-complex protein 1 subunit beta or Chaperonin containing TCP1 Subunit 2	Molecular chaperone protein complex involved with the folding of proteins upon ATP hydrolysis	Functions as an aggrephagy receptor promoting the clearance of solid protein aggregates, including tau [1] Significantly downregulated in AD, including in the hippocampus and temporal cortex [2]
	CCT4	T-complex protein 1 subunit delta Or Chaperonin containing TCP1 Subunit 4	Molecular chaperone protein complex involved with the folding of proteins upon ATP hydrolysis	--
	CCT5	T-complex protein 1 subunit epsilon or Chaperonin containing TCP1 Subunit 5	Molecular chaperone protein complex involved with the folding of proteins upon ATP hydrolysis	Promotes tau phosphorylation and detachment from microtubules in a CDK-5-dependent manner and concurrently improves retrograde axonal BDNF transport function [3]
	EFTUD2	116 kDa U5 Small Nuclear Ribonucleoprotein Component or Elongation Factor Tu GTP Binding Domain Containing 2	GTPase component of the spliceosome complex involved in pre-mRNA splicing	--
	HSPB1	Heat Shock Protein Beta-1 Or Heat Shock Protein 27	Molecular chaperone protein involved in promoting the correct folding of proteins, stress resistance, and actin organization	Prevents tau filament formation and aggregation[4,5], aids in tau clearance, rescues LTP, [4, 6] synaptic dysfunction, [5] and learning and memory deficits, and reduces amyloid plaque burden [6]
	NACA	Nascent Polypeptide-associated Complex subunit alpha	Binds to nascent proteins lacking a signal peptide motif blocking the interaction with the signal recognition particle and prevents mistranslocation to the endoplasmic reticulum	Suppresses Aβ40 aggregation and fibril formation *in vitro* [7]

	PABPC1	Polyadenylate-Binding Protein 1<br>Or<br>Poly(A) Binding Protein Cytoplasmic 1	Binds to the poly(A) tail of mRNA and modulates pre-mRNA splicing, mRNA stability, and mRNA decay	Interacts with tau(1-144) [8]
	PSMC2	26S Proteasome Regulatory Subunit 7<br>Or<br>Proteasome 26S Subunit, ATPase 2	ATPase subunit that has chaperone-like activity involved in ATP-dependent degradation of ubiquitinated proteins and may participate in the regulation of transcription	Activates NF1 and is co-expressed and downregulated with NF1 in the hippocampus in AD [9]<br>Mediates neuroinflammation by increasing NF-κB activation through IκBα degradation and increases the production of pro-inflammatory NO and PGE_2, through upregulating iNOS and COX-2 expression, respectively [10]
	PSMD14	26S Proteasome non-ATPase Regulatory Subunit 14	A metalloprotease subunit involved in the cleavage of 'Lys-63'-linked polyubiquitin chains, regulates non-homologous end joining (NHEJ) and homologous repair and responds to double-stranded breaks	Downregulated in multiple brain regions in AD patient brain samples, including the hippocampus, entorhinal cortex[11], and AD iPSCs [12]
	RPL7A	60S Ribosomal Protein L7a	A ribosomal protein involved in the synthesis of proteins, it can interact with nuclear hormone receptors binding to their DNA response elements inhibiting their ability to transactivate	Significantly upregulated in the neocortex and corona radiata brain capillaries of AD patients [13]
	RPL8	60S Ribosomal Protein L8	A ribosomal involved in the synthesis of proteins and is a constituent of the elongation factor 2-binding site at the ribosomal subunit interface	Significantly upregulated in the neocortex and corona radiata brain capillaries of AD patients [13]<br>Significantly upregulated in APP/PS1 and Ferroportin1-floxed murine models of AD [14]
	RPL10A	60S Ribosomal Protein L10a<br>Or<br>NEDD6	A ribosomal protein involved in the synthesis of proteins	Significantly upregulated in the neocortex and corona radiata brain capillaries of AD patients [13]<br>Significantly downregulated in AD blood samples [15]
	RPL23	60S Ribosomal Protein L23<br>Or<br>RPL17	A ribosomal protein involved in the synthesis of proteins	Significantly upregulated in the neocortex brain capillaries of AD patients[13]<br>Significantly downregulated in AD blood samples [15], and in the tau frontotemporal dementia mouse model [16]

	RPLP0	60S Acidic Ribosomal Protein P0<br>Or<br>Ribosomal Protein Lateral Stalk Subunit P0	Functionally equivalent to the E.coli L10 ribosomal protein	Significantly upregulated in the neocortex and corona radiata brain capillaries of AD patients [13]<br>Significantly decreased in tau frontotemporal dementia mouse model [16]
	RPLP2	60S Acidic Ribosomal Protein P2<br>Or<br>Ribosomal Protein Lateral Stalk Subunit P2	Functionally equivalent to the E.coli L7/L12 ribosomal protein and involved in the elongation step of protein synthesis	--
	RPS3A	40S Ribosomal Protein S3a<br>Or<br>MFTL	A ribosomal protein that may play a role in erythropoiesis	Significantly upregulated in the neocortex and corona radiata brain capillaries of AD patients [13]<br>Significantly downregulated in AD blood samples [15]
	RPS6	40S Ribosomal Protein S6	Ribosomal protein that is a substrate of protein kinases; it also plays a role in regulating cell growth and proliferation	Significantly downregulated in AD blood, in the dentate gyrus (Braak stages I-IV), and in the CA1 (Braak stages I-II;V-VI) [17]<br>pRPS6 localizes to granulovacuolar degeneration within pyramidal neurons and may indicate an adaptive neuroprotective mechanism in AD [18]
	RPS8	40S Ribosomal Protein S6	A ribosomal protein	Significantly upregulated in the corona radiata brain capillaries of AD patients [13]<br>Significantly downregulated in AD blood samples [15]
	RPS16	40S Ribosomal Protein S16	A ribosomal protein	Significantly upregulated in the corona radiata brain capillaries of AD patients [13]<br>Significantly decreased in tau frontotemporal dementia mouse model [16], and in the dentate gyrus and CA1 of late stage AD patients (Braak stages V-VI) [17]
	RPS23	40S Ribosomal Protein S23	A ribosomal protein involved with protein synthesis, RNA binding, and translational accuracy	Retroposed RPS23 mRNA reduces β-amyloid levels and tau phosphorylation by activating the adenylate cyclase 8/cAMP/PKA pathway enhancing CREB and inhibiting GSK-3 activity, [19, 20] and

				is important in maintaining synaptic plasticity [20, 21]
	RPS25	40S Ribosomal Protein S25	A ribosomal protein involved with RNA binding	Significantly upregulated in the corona radiata brain capillaries of AD patients [13]
	RPSA	40S Ribosomal Protein SA<br>Or<br>LAMR1	Needed for the assembly and stability of the 40S ribosomal subunit, it additionally functions as a laminin receptor, plays a role in cell adhesion, and activates signaling transduction pathways	Significantly upregulated in the neocortex and corona radiata brain capillaries of AD patients [13]<br>Affects APP maturation, possibly through interactions with ɣ- and β-secretase [22, 23], Aβ shedding [23], and may contribute to mitochondrial dysfunction [22]
	STIP1	Stress Induced Phosphoprotein 1<br>Or<br>Hop	Co-chaperone of Hsp70 and Hsp90, as it coordinates the protein folding functions and works to stimulate the ATPase activity of HSP70 and inhibits the ATPase activity of HSP90	Hsp70:Hop:HSP90 complex may be a critical regulator of tau homeostasis; it inhibits and delays tau fibrillization; however, it may have a harmful holding function of ptau [24]<br>Directly able to inhibit Aβ binding to PrP^{C}, thereby rescuing neuronal cell death and alleviating synaptic loss [25, 26]
	TCP1	T-complex Protein 1 Subunit Alpha	A component of the chaperonin containing T-complex (TRiC), works to fold proteins upon ATP-hydrolysis, regulates telomere maintenance, and is involved in ciliogenesis.	TCP-1/β 1 tubulin ratio significantly decreased in AD [27]<br>TCP1 in the TRiC/CCT complex may mediate Aβ toxicity [28]
**Group 2**	ACTA1	Actin Alpha 1, Skeletal Muscle	Plays a role in cell motility, structure, and integrity	--
	ACTN1	Actinin Alpha 1	A bundling protein that in non-muscle cells works to bind actin to the membrane; in muscle cells, it helps to anchor the myofibrillar actin filaments	Aβ may reduce phosphorylation levels of ACTN1 [29]<br>Decreased in AD [30]
	ACTN4	Actinin Alpha 4	A bundling protein that in non-muscle cells works to bind actin to the membrane; in muscle cells, it helps to anchor the myofibrillar actin filaments and may also be involved in vesicular trafficking	Higher levels of ACTN4 in AD patients are significantly associated with resilience against AD [31]<br>Dysregulated in APP murine models [32, 33]
	BSG	Basigin<br>Or<br>CD147	A member of the immunoglobulin family, as well as a plasma membrane protein that is involved in spermatogenesis, neural network formation, and tumor progression	An integral regulatory subunit of the γ-secretase complex, which upon depletion, upregulates the production of Aβ peptides [34]

				Hypoxia enhances the interaction of CD147 and Hook1, increasing Aβ in exosomes [35]
	CAPN1	Calpain-1 catalytic subunit	A calcium-regulated non-lysosomal intracellular cysteine protease known to catalyze substrates involved in cytoskeletal remodeling and signal transduction	Cleaves both normal tau and a pathogenic fragment of tau [36]<br>Important for neuronal survival, synaptic plasticity, learning, and memory, and may be neuroprotective against AD [37]<br>Significantly correlated with increased Aβ (1-42), α-spectrin, p25, and occurring alongside activation of CDK-5 and GSK-3 [38]
	CTNNA1	Catenin alpha-1	Plays a role in cell adhesion through binding cadherins to actin filaments	--
	CTNND1	Catenin delta-1	Regulates the C-, E-, and N-cadherins properties facilitating cell adhesion, as well as cell transformation, and ligand-induced receptor signaling	The target of hsa-miR-1205 and hsa-miR-425-5p in late-stage AD [39]
	CYFIP2	Cytoplasmic FMR1 Interacting Protein 2	Possesses small GTPase biding ability involved in apoptosis, cell-cell adhesion, a component of the WAVE complex, and is necessary for BDNF-NTRK2 endocytic trafficking, and signaling	Significantly decreased in late-stage AD patients' hippocampus and superior temporal gyrus, the reduction causes an increase in APP, BACE-1, and CAMKII protein expression, increasing Aβ(1-42) and hyperphosphorylated tau, respectively [40]<br>Aβ(1-42) may play a role in reducing CYFIP2 expression through activation of Mnk1-eLF4E-CYFIP axis, contributing to neuropathic changes, dendritic spine loss, and impaired hippocampus-memory formation [41]
	DAG1	Dystroglycan 1	Component of the dystrophin-glycoprotein complex important for numerous functions such as laminin and basement membrane assembly, sarcolemma stability, cell survival, cell migration, and epithelial polarization	Significantly associated with dementia status and phosphorylated tau in the temporal cortex [42]
	FYN	Tyrosine-protein Kinase Fyn<br>Or<br>FYN Proto-Oncogene, Src Family Tyrosine Kinase	Membrane-bound tyrosine kinase plays a role in many functions, such as cell growth, cell adhesion, cytoskeletal remodeling, cell motility, immune and axon guidance	Mediates signal transduction from the Aβ-PrP^c complex; Aβ activates the PrP^c/FYN pathway leading to phosphorylation of the NR2B subunit of NMDARs, [43] and dendritic spine loss through mGLUR5 interaction [44]

				Inhibition of FYN in the AD mouse model rescues memory deficits, and synaptic plasticity, reduces microgliosis, and limits tau aggregation [45]
	LAMTOR3	Ragulator Complex Protein LAMTOR3<br>Or<br>Late Endosomal/Lysosomal Adaptor, MAPK And MTOR Activator 3	Scaffolding protein that is a part of the Ragulator complex involved in the extracellular signal-regulated kinase (ERK) cascade, amino acid sensing, and mTORC1 activation	Elevated in old age female vervets [46]
	MAPK1	Mitogen-activated Protein Kinase 1<br>Or<br>ERK2	MAP kinase, otherwise known as extracellular signal-regulated kinases (ERKs) involved in multitudinous functions such as proliferation, differentiation, transcription regulation, development, and cytoskeletal rearrangement	ERK1/2 pathway is involved in multitudinous functions in AD, including regulating Aβ production through differing interactions with α-secretase, BACE1, and γ-secretase, and Aβ clearance, tau phosphorylation through activation of p53, CDK-5, and GSK-3, which may mediate PP2A activity, neuroinflammation through AP-1 and NF-κB activation, oxidative stress, and neural loss [47]
	MAPK3	Mitogen-activated Protein Kinase 3<br>Or<br>ERK1	MAP kinase, otherwise known as extracellular signal-regulated kinases (ERKs) involved in multitudinous functions such as proliferation, differentiation, transcription regulation, development, and cytoskeletal rearrangement	ERK1/2 pathway involved in multitudinous functions in AD including regulating Aβ production through differing interactions with α-secretase, BACE1, and γ-secretase, and Aβ clearance, tau phosphorylation through activation of p53, CDK-5, and GSK-3, which may mediate PP2A activity, neuroinflammation through AP-1 and NF-κB activation, oxidative stress, and neural loss [47]
	NCKAP1	Nck-associated protein 1	A component of the SCAR/WAVE complex, involved in the regulation of actin filament organization, important for BDNF-NTRK2 endocytic trafficking, signaling, and GTPase binding	Significantly decreased in female sporadic AD patients [48]
	PEA15	Astrocytic phosphoprotein PEA-15<br>or	Death effector domain-containing protein that is important for the negative regulation of apoptosis, it is also an endogenous substrate of	Aβ upregulates PEA15 expression and is shown to participate in astrocyte-mediated phagocytosis of Aβ [49]

		Proliferation And Apoptosis Adaptor Protein 15	protein kinase C, and regulates glucose transport	Localized to reactive astrocytes around neocortical amyloid plaques in human and mouse models [50]<br>Shields astrocytes from TNFα-induced apoptosis, possibly through binding to DED-containing protein FADD and caspase-8 [51]
	PFN1	Profilin 1	Small actin-binding protein that is important in actin dynamics such as actin polymerization, and affects cytoskeleton structure	Decreased levels may play a role in the impairment of synaptic plasticity and spatial memory in aged APP/PS1 mouse model of AD [52, 53, 54]
	RAC1	Ras-related C3 botulinum toxin substrate 1<br>Or<br>Rac Family Small GTPase 1	A plasma-membrane associated GTPase involved in multitudinous functions such as regulation of cell growth, cytoskeletal reorganization, and activation of protein kinases, including in its GTP-bound state regulation of secretory processes, phagocytosis, neuronal adhesion, migration, and differentiation	Increased activity in both patients and animal models of AD, Aβ42 upregulates RAC1 expression, which leads to accelerated memory loss and spatial decay, which is ameliorated upon RAC1 inhibition [55]<br>Knockdown of RAC1 in neurons of *Drosophila* resulted in age-dependent behavioral deficits and neurodegeneration [56]
	RAP1B	RAP1B, Member Of RAS Oncogene Family<br>Or<br>Ras-related protein Rap-1b	RAS-like small GTP-binding protein with GTPase activity involved in cell adhesion, growth, differentiation, and basal endothelial barrier function; it may also regulate integrin-mediated cell signaling.	--
	RHOA	Ras Homolog Family Member A<br>Or<br>Transforming protein RhoA	A small GTPase important in signal transduction cascades, cytoskeleton organization, dynamics, cell migration, and cycle, as well as linking plasma membrane receptors to the assembly of focal adhesions and actin stress fibers	Upregulation of RhoA/ROCK signaling pathway activation increases Aβ expression through increasing β- and ɣ-secretase activity, tau hyperphosphorylation, and affects tau's ability to bind to tubulin; Aβ can activate the RhoA/ROCK pathway activating microglia and inducing neuroinflammation and neural apoptosis [57]
	ROBO1	Roundabout Homolog 1<br>or<br>Roundabout Guidance Receptor 1	An integral membrane protein that is a receptor for SLIT1 and SLIT2 and functions in cellular migration, including axonal guidance and neuronal precursor migration	SLIT/ROBO pathway regulates PAK2 activity directly, as well as activates SRGAP1 protein, which attenuates PAK2 activity, both of which may lead to alterations in synaptic morphology and axonal migration, and may lead to neuronal death [54]

	ROBO2	Roundabout Homolog 2<br>or<br>Roundabout Guidance Receptor 2	A transmembrane receptor that is a receptor for SLIT2 and functions in cellular migration, including axonal guidance and neuronal development	SLIT/ROBO pathway regulates PAK2 activity, as well as activates SRGAP1 protein, which attenuates PAK2 activity, both of which may lead to alterations in synaptic morphology and axonal migration, and may lead to neuronal death [54]
	SRC	Proto-oncogene tyrosine-protein kinase Src	A non-receptor tyrosine kinase involved in numerous functions such as gene transcription, immune response, cell adhesion, cell cycle progression, apoptosis, migration, and transformation	May mediate CHGA-activated microglial apoptotic cascade through ERK1/2 phosphorylation leading to increase iNOS expression and NO production [58]<br>Involved in the activation of microglia and increased TNFα in response to Aβ fibrils *in vivo* and *in vitro* [59]
	TLN1	Talin 1	Cytoskeletal protein involved in actin filament assembly, spreading, and activation of fibroblasts and osteoclasts	--
	WASF2	WASP Family Member 2<br>Or<br>Actin-binding protein WASF2	A downstream effector molecule that is part of a multiprotein WAVE complex involved in the transmission of signals from tyrosine kinase receptors and small GTPases to the actin cytoskeleton	--
	YES1	YES Proto-Oncogene 1, Src Family Tyrosine Kinase	A non-receptor tyrosine kinase involved in numerous functions such as cell growth and survival, cell-cell adhesion, apoptosis, cytoskeleton remodeling, and differentiation	--
**Group 3**	BGN	Biglycan	Member of the small-leucine-rich proteoglycan (SLRP) family of proteins that may be involved in bone growth, muscle development and regeneration, inflammation, innate immunity, and collagen fiber assembly	Positively associated with apo B lipoprotein within amyloid plaque core [60]
	BMP1	Bone Morphogenetic Protein 1<br>Or<br>PCOLC	Metalloprotease involved in regulation of the extracellular matrix, cartilage formation, bone formation, muscle growth and homeostasis, and wound healing	--
	CD14	Monocyte differentiation antigen CD14	Surface antigen expressed on monocytes and macrophages involved in the innate immune system and is	Interacts with fibrillar Aβ(1-42), [61] plays a role in $Aβ_{42}$ phagocytosis [62], is involved in Aβ induced-microglial

			known to mediate the response to bacterial lipopolysaccharide (LPS)	activation and associated neurotoxicity [61, 63], and may modulate *Tnfα, Il-10,* and *Ym1* [63]
	COL1A1	Collagen Alpha-1(I) Chain	Fibril forming collagen	--
	COL2A1	Collagen Alpha-1(II) Chain	Fibrillar collagen important for the normal embryonic development of the skeleton, linear growth, and the ability of cartilage to resist compressive forces	Altered in neurons derived from patients with PSEN1, PSEN2, and APOE4 mutations, higher methylation levels were significantly associated with decreased risk of death [64]
	COL5A1	Collagen Alpha-1(V) Chain	Fibril-forming collagen that may regulate the assembly of heterotypic fibers	--
	COL5A2	Collagen Alpha-2(V) Chain	Fibril-forming collagen that may regulate the assembly of heterotypic fibers and tissue-specific matrices	Downregulated in AD [65]
	COL5A3	Collagen Alpha-3(V) Chain	Fibril-forming collagen that may regulate the assembly of heterotypic fibers	--
	COL6A1	Collagen Alpha-1(VI) Chain	Cell binding protein that is a structural component of microfibrils	Increased levels in the dentate gyrus of AD patients; may prevent Aβ42 neurotoxicity through extracellular sequestration [66].<br>Found in EVs of CTE football players [74].
	COL6A2	Collagen Alpha-2(VI) Chain	Cell binding protein binds to extracellular matrix proteins	May be a selective marker for cerebral amyloid angiopathy in AD patients [67]
	COL6A3	Collagen Alpha-3(VI) Chain	Cell binding protein binds to extracellular matrix proteins	Significantly increased in AD patients with cerebral amyloid angiopathy [67]<br>Found in EVs of CTE football players [74].
	COL11A1	Collagen Alpha-1(XI) Chain	A structural constituent of the extracellular matrix and may be involved in fibrillogenesis	--
	COL11A2	Collagen Alpha-2(XI) Chain	A structural constituent of the extracellular matrix that gives tensile strength; may be involved in fibrillogenesis	--
	COL14A1	Collagen Alpha-1(XIV) Chain	Integrates collagen bundles, is involved with RNA binding, and is a structural constituent of the extracellular matrix	Altered in neurons derived from patients with PSEN1, PSEN2, and APOE4 mutations; higher methylation levels were significantly associated with decreased risk of death [64]

	DCN	Decorin	Small leucine-rich proteoglycan protein involved in collagen fibril assembly	Localizes to the edges of amyloid plaques and amyloid fibril bundles [68]<br>Positively associated with apo B lipoprotein within amyloid plaque core and edges [60]
	FBN1	Fibrillin-1	An extracellular matrix glycoprotein important in calcium-binding microfibrils, long-term force-bearing structural support, and tissue homeostasis	miR-29a is decreased in AD, of which FBN1 is a target [69]
	FBN2	Fibrillin-2	Connective tissue microfibril that may regulate elastic fiber assembly	Significantly decreased following Aβ treatment *in vitro* [70]
	PCOLCE	Procollagen C-Endopeptidase Enhancer<br>Or<br>PCPE1	Glycoprotein is involved in binding and driving cleavage of type 1 procollagen and upregulating procollagen C-proteinase activity	May participate in amyloidogenesis [71, 72]
	POSTN	Periostin	Secreted extracellular matrix protein involved in tissue development and regeneration, cell adhesion, and wound healing	--
	SPARC	Secreted Protein Acidic And Cysteine Rich<br>Or<br>ON	Cysteine-rich acidic matrix-associated protein involved in extracellular matrix synthesis and cell growth regulation through cytokine and extracellular matrix interaction	Upregulated in the AD brain and shown to colocalize to Aβ protein deposits; may be involved in may be involved in the immune response to neuropathological features of AD [73]
	TLL1	Tolloid Like 1	A metalloprotease involved in processing procollagen C-pro-peptides, such as pro-biglycan	--

References:

1. Ma X, Lu C, Chen Y, et al. CCT2 is an aggrephagy receptor for clearance of solid protein aggregates. *Cell*. 2022 Apr 14;185(8):1325-1345.e22. [PMID: 35366418 DOI: 10.1016/j.cell.2022.03.005]

2. Ma X, Feng Y, Quan X, et al. Multi-omics analysis revealed the role of CCT2 in the induction of autophagy in Alzheimer's disease. *Front Genet.* 2023 Jan 10;13:967730. [PMID: 36704351 DOI: 10.3389/fgene.2022.967730]

3. Chen XQ, Fang F, Florio JB, et al. T-complex protein 1-ring complex enhances retrograde axonal transport by modulating tau phosphorylation. *Traffic*. 2018 Nov;19(11):840-853. [PMID: 30120810 DOI: 10.1111/tra.12610]

4. Abisambra JF, Blair LJ, Hill SE, et al. Phosphorylation dynamics regulate Hsp27-mediated rescue of neuronal plasticity deficits in tau transgenic mice. *J Neurosci.* 2010 Nov 17;30(46):15374-82. [PMID: 21084594 DOI: 10.1523/JNEUROSCI.3155-10.2010]

5. Zhang S, Zhu Y, Lu J, et al. Specific binding of Hsp27 and phosphorylated Tau mitigates abnormal Tau aggregation-induced pathology. *Elife*. 2022 Sep 1;11:e79898. [PMID: 36048712 DOI: 10.7554/eLife.79898]

6. Tóth ME, Szegedi V, Varga E, et al. Overexpression of Hsp27 ameliorates symptoms of Alzheimer's disease in APP/PS1 mice. *Cell Stress Chaperones*. 2013 Nov;18(6):759-71. [PMID: 23605646 DOI: 10.1007/s12192-013-0428-9].

7. Shen K, Gamerdinger M, Chan R, et al. Dual Role of Ribosome-Binding Domain of NAC as a Potent Suppressor of Protein Aggregation and Aging-Related Proteinopathies. *Mol Cell.* 2019 May 16;74(4):729-741.e7. [PMID: 30982745 DOI: 10.1016/j.molcel.2019.03.012]

8. Gunawardana CG, Mehrabian M, Wang X, et al. The Human Tau Interactome: Binding to the Ribonucleoproteome, and Impaired Binding of the Proline-to-Leucine Mutant at Position 301 (P301L) to Chaperones and the Proteasome. *Mol Cell Proteomics*. 2015 Nov;14(11):3000-14. [PMID: 26269332 DOI: 10.1074/mcp.M115.050724]

9. Dharshini SAP, Taguchi YH, Gromiha MM. Investigating the energy crisis in Alzheimer disease using transcriptome study. *Sci Rep*. 2019 Dec 6;9(1):18509. [PMID: 31811163 DOI: 10.1038/s41598-019-54782-y]

10. Bi W, Jing X, Zhu L, et al. Inhibition of 26S protease regulatory subunit 7 (MSS1) suppresses neuroinflammation. *PLoS One*. 2012;7(5):e36142. [PMID: 22629310 DOI: 10.1371/journal.pone.0036142]

11. Puthiyedth N, Riveros C, Berretta R, Moscato P. Identification of Differentially Expressed Genes through Integrated Study of Alzheimer's Disease Affected Brain Regions. *PLoS One*. 2016 Apr 6;11(4):e0152342. [PMID: 27050411 DOI: 10.1371/journal.pone.0152342]

12. Hossini AM, Megges M, Prigione A, et al. Induced pluripotent stem cell-derived neuronal cells from a sporadic Alzheimer's disease donor as a model for investigating AD-associated gene regulatory networks. *BMC Genomics*. 2015 Feb 14;16(1):84. [PMID: 25765079 DOI: 10.1186/s12864-015-1262-5] Erratum in: *BMC Genomics*. 2015;16:433 [PMID: 25765079].

13. Suzuki M, Tezuka K, Handa T, et al. Upregulation of ribosome complexes at the blood-brain barrier in Alzheimer's disease patients. J Cereb *Blood Flow Metab*. 2022 Nov;42(11):2134-2150. [PMID: 35766008 DOI: 10.1177/0271678X221111602]

14. Bao WD, Pang P, Zhou XT, et al. Loss of ferroportin induces memory impairment by promoting ferroptosis in Alzheimer's disease. *Cell Death Differ*. 2021 May;28(5):1548-1562. [PMID: 33398092 DOI: 10.1038/s41418-020-00685-9]

15. Shigemizu D, Mori T, Akiyama S, et al. Identification of potential blood biomarkers for early diagnosis of Alzheimer's disease through RNA sequencing analysis. *Alzheimers Res Ther*. 2020 Jul 16;12(1):87. [PMID: 32677993 DOI: 10.1186/s13195-020-00654-x]

16. Evans HT, Benetatos J, van Roijen M, Bodea LG, Götz J. Decreased synthesis of ribosomal proteins in tauopathy revealed by non-canonical amino acid labelling. *EMBO J*. 2019 Jul 1;38(13):e101174. [PMID: 31268600 DOI: 10.15252/embj.2018101174]

17. Hernández-Ortega K, Garcia-Esparcia P, Gil L, Lucas JJ, Ferrer I. Altered Machinery of Protein Synthesis in Alzheimer's: From the Nucleolus to the Ribosome. *Brain Pathol*. 2016 Sep;26(5):593-605. [PMID: 26512942 DOI: 10.1111/bpa.12335]

18. Castellani RJ, Gupta Y, Sheng B, et al. A novel origin for granulovacuolar degeneration in aging and Alzheimer's disease: parallels to stress granules. *Lab Invest*. 2011 Dec;91(12):1777-86. [PMID: 21968813 DOI: 10.1038/labinvest.2011.149]

19. Huang X, Chen Y, Li WB, et al. The Rps23rg gene family originated through retroposition of the ribosomal protein s23 mRNA and encodes proteins that decrease Alzheimer's beta-amyloid level and tau phosphorylation. *Hum Mol Genet*. 2010 Oct 1;19(19):3835-43. [PMID: 20650958 DOI: 10.1093/hmg/ddq302]

20. Yan L, Chen Y, Li W, et al. RPS23RG1 reduces Aβ oligomer-induced synaptic and cognitive deficits. *Sci Rep*. 2016 Jan 6;6:18668. [PMID: 26733416 DOI: 10.1038/srep18668]

21. Zhao D, Meng J, Zhao Y, et al. RPS23RG1 Is Required for Synaptic Integrity and Rescues Alzheimer's Disease-Associated Cognitive Deficits. *Biol Psychiatry*. 2019 Aug 1;86(3):171-184. [PMID: 30292394 doi: 10.1016/j.biopsych.2018.08.009]

22. Bhattacharya A, Izzo A, Mollo N, et al. Inhibition of 37/67kDa Laminin-1 Receptor Restores APP Maturation and Reduces Amyloid-β in Human Skin Fibroblasts from Familial Alzheimer's Disease. *J Pers Med*. 2020 Nov 16;10(4):232. [PMID: 33207563 DOI: 10.3390/jpm10040232]

23. Jovanovic K, Loos B, Da Costa Dias B, Penny C, Weiss SF. High resolution imaging study of interactions between the 37 kDa/67 kDa laminin receptor and APP, beta-secretase and gamma-secretase in Alzheimer's disease. *PLoS One*. 2014 Jun 27;9(6):e100373. [PMID: 24972054 DOI: 10.1371/journal.pone.0100373]

24. Moll A, Ramirez LM, Ninov M, Schwarz J, Urlaub H, Zweckstetter M. Hsp multichaperone complex buffers pathologically modified Tau. *Nat Commun*. 2022 Jun 27;13(1):3668. [PMID: 35760815 DOI: 10.1038/s41467-022-31396-z]

25. Maciejewski A, Ostapchenko VG, Beraldo FH, Prado VF, Prado MA, Choy WY. Domains of STIP1 responsible for regulating PrPC-dependent amyloid-β oligomer toxicity. *Biochem J*. 2016 Jul 15;473(14):2119-30. [PMID: 27208175 DOI: 10.1042/BCJ20160087]

26. Ostapchenko VG, Beraldo FH, Mohammad AH, et al. The prion protein ligand, stress-inducible phosphoprotein 1, regulates amyloid-β oligomer toxicity. *J Neurosci*. 2013 Oct 16;33(42):16552-64. [DOI: 10.1523/JNEUROSCI.3214-13.2013] Erratum in: *J Neurosci*. 2015 Jan 28;35(4):1816. [PMID: 24133259]

27. Schuller E, Gulesserian T, Seidl R, Cairns N, Lube G. Brain t-complex polypeptide 1 (TCP- 1) related to its natural substrate beta1 tubulin is decreased in Alzheimer's disease. *Life Sci.* 2001 Jun 8;69(3):263-70. [PMID: 11441917 DOI: 10.1016/s0024-3205(01)01126-2]

28. Khabirova E, Moloney A, Marciniak SJ, et al. The TRiC/CCT chaperone is implicated in Alzheimer's disease based on patient GWAS and an RNAi screen in Aβ-expressing Caenorhabditis elegans. *PLoS One*. 2014 Jul 31;9(7):e102985. [PMID: 25080104 DOI: 10.1371/journal.pone.0102985]

29. Henriques AG, Müller T, Oliveira JM, Cova M, da Cruz E Silva CB, da Cruz E Silva OA. Altered protein phosphorylation as a resource for potential AD biomarkers. *Sci Rep*. 2016 Jul 28;6:30319. [PMID: 27466139 DOI: 10.1038/srep30319]

30. Hondius DC, van Nierop P, Li KW, et al. Profiling the human hippocampal proteome at all pathologic stages of Alzheimer's disease. *Alzheimers Dement*. 2016 Jun;12(6):654-68. [PMID: 26772638 DOI: 10.1016/j.jalz.2015.11.002]

31. Yu L, Tasaki S, Schneider JA, et al. Cortical Proteins Associated With Cognitive Resilience in Community-Dwelling Older Persons. *JAMA Psychiatry*. 2020 Nov 1;77(11):1172-1180. [PMID: 32609320 DOI: 10.1001/jamapsychiatry.2020.1807]

32. Badhwar A, Brown R, Stanimirovic DB, Haqqani AS, Hamel E. Proteomic differences in brain vessels of Alzheimer's disease mice: Normalization by PPARγ agonist pioglitazone. *J Cereb Blood Flow Metab*. 2017 Mar;37(3):1120-1136. [PMID: 27339263 DOI: 10.1177/0271678X16655172]

33. Muraoka S, Jedrychowski MP, Iwahara N, et al. Enrichment of Neurodegenerative Microglia Signature in Brain-Derived Extracellular Vesicles Isolated from Alzheimer's Disease Mouse Models. *J Proteome Res*. 2021 Mar 5;20(3):1733-1743. [PMID: 33534581 DOI: 10.1021/acs.jproteome.0c00934]

34. Zhou S, Zhou H, Walian PJ, Jap BK. CD147 is a regulatory subunit of the gamma-secretase complex in Alzheimer's disease amyloid beta-peptide production. *Proc Natl Acad Sci USA*. 2005 May 24;102(21):7499-504. [PMID: 15890777 DOI: 10.1073/pnas.0502768102]

35. Xie JC, Ma XY, Liu XH, et al. Hypoxia increases amyloid-β level in exosomes by enhancing the interaction between CD147 and Hook1. *Am J Transl Res*. 2018 Jan 15;10(1):150-163. [PMID: 29423001]

36. Chen HH, Liu P, Auger P, et al. Calpain-mediated tau fragmentation is altered in Alzheimer's disease progression. *Sci Rep.* 2018 Nov 13;8(1):16725. [PMID: 30425303 DOI: 10.1038/s41598-018-35130-y]

37. Su W, Zhou Q, Wang Y, et al. Deletion of the *Capn1* Gene Results in Alterations in Signaling Pathways Related to Alzheimer's Disease, Protein Quality Control and Synaptic Plasticity in Mouse Brain. *Front Genet.* 2020 Apr 9;11:334. [PMID: 32328086 DOI: 10.3389/fgene.2020.00334]

38. Kurbatskaya K, Phillips EC, Croft CL, et al. Upregulation of calpain activity precedes tau phosphorylation and loss of synaptic proteins in Alzheimer's disease brain. *Acta Neuropathol Commun.* 2016 Mar 31;4:34. [PMID: 27036949 DOI: 10.1186/s40478-016-0299-2]

39. Watson CN, Begum G, Ashman E, et al. Co-Expression Analysis of microRNAs and Proteins in Brain of Alzheimer's Disease Patients. *Cells*. 2022 Jan 4;11(1):163. [PMID: 35011725 DOI: 10.3390/cells11010163]

40. Tiwari SS, Mizuno K, Ghosh A, et al. Alzheimer-related decrease in CYFIP2 links amyloid production to tau hyperphosphorylation and memory loss. *Brain*. 2016 Oct;139(Pt 10):2751-2765. [PMID: 27524794 DOI: 10.1093/brain/aww205]

41. Ghosh A, Mizuno K, Tiwari SS, et al. Alzheimer's disease-related dysregulation of mRNA translation causes key pathological features with ageing. *Transl Psychiatry*. 2020 Jun 16;10(1):192. [PMID: 32546772 DOI: 10.1038/s41398-020-00882-7]

42. Simon MJ, Wang MX, Murchison CF, et al. Transcriptional network analysis of human astrocytic endfoot genes reveals region-specific associations with dementia status and tau pathology. *Sci Rep.* 2018 Aug 17;8(1):12389. [PMID: 30120299 DOI: 10.1038/s41598-018-30779-x]

43. Um JW, Nygaard HB, Heiss JK, et al. Alzheimer amyloid-β oligomer bound to postsynaptic prion protein activates Fyn to impair neurons. *Nat Neurosci.* 2012 Sep;15(9):1227-35. [PMID: 22820466 DOI: 10.1038/nn.3178]

44. Um JW, Kaufman AC, Kostylev M, et al. Metabotropic glutamate receptor 5 is a coreceptor for Alzheimer aβ oligomer bound to cellular prion protein. *Neuron*. 2013 Sep 4;79(5):887-902. [DOI: 10.1016/j.neuron.2013.06.036] Erratum in: *Neuron.* 2013 Oct 16;80(2):531. [PMID: 24012003]

45. Kaufman AC, Salazar SV, Haas LT, et al. Fyn inhibition rescues established memory and synapse loss in Alzheimer mice. *Ann Neurol*. 2015 Jun;77(6):953-71. [PMID: 25707991 DOI: 10.1002/ana.24394]

46. Negrey JD, Dobbins DL, Howard TD, et al. Transcriptional profiles in olfactory pathway-associated brain regions of African green monkeys: Associations with age and Alzheimer's disease neuropathology. *Alzheimers Dement (N Y)*. 2022 Oct 27;8(1):e12358. [PMID: 36313967 DOI: 10.1002/trc2.12358]

47. Khezri MR, Yousefi K, Esmaeili A, Ghasemnejad-Berenji M. The Role of ERK1/2 Pathway in the Pathophysiology of Alzheimer's Disease: An Overview and Update on New Developments. *Cell Mol Neurobiol*. 2023 Jan;43(1):177-191. [PMID: 35038057 DOI: 10.1007/s10571-022-01191-x]

48. Suzuki T, Nishiyama K, Yamamoto A, et al. Molecular cloning of a novel apoptosis-related gene, human Nap1 (NCKAP1), and its possible relation to Alzheimer disease. *Genomics.* 2000 Jan 15;63(2):246-54. [PMID: 10673335 DOI: 10.1006/geno.1999.6053]

49. Lv J, Ma S, Zhang X, et al. Quantitative proteomics reveals that PEA15 regulates astroglial Aβ phagocytosis in an Alzheimer's disease mouse model. *J Proteomics*. 2014 Oct 14;110:45-58. [PMID: 25108202 DOI: 10.1016/j.jprot.2014.07.028]

50. Thomason LA, Smithson LJ, Hazrati LN, McLaurin J, Kawaja MD. Reactive astrocytes associated with plaques in TgCRND8 mouse brain and in human Alzheimer brain express phosphoprotein enriched in astrocytes (PEA-15). *FEBS Lett*. 2013 Aug 2;587(15):2448-54. [PMID: 23792157 DOI: 10.1016/j.febslet.2013.06.015]

51. Kitsberg D, Formstecher E, Fauquet M, et al. Knock-out of the neural death effector domain protein PEA-15 demonstrates that its expression protects astrocytes from TNFalpha-induced apoptosis. *J Neurosci*. 1999 Oct 1;19(19):8244-51. [PMID: 10493725 DOI: 10.1523/JNEUROSCI.19-19-08244.1999]

52. Lian B, Liu M, Lan Z, et al. Hippocampal overexpression of SGK1 ameliorates spatial memory, rescues Aβ pathology and actin cytoskeleton polymerization in middle-aged APP/PS1 mice. *Behav Brain Res*. 2020 Apr 6;383:112503. [PMID: 31981651 DOI: 10.1016/j.bbr.2020.112503]

53. Sun H, Liu M, Sun T, et al. Age-related changes in hippocampal AD pathology, actin remodeling proteins and spatial memory behavior of male APP/PS1 mice. *Behav Brain Res*. 2019 Dec 30;376:112182. [PMID: 31472195 DOI: 10.1016/j.bbr.2019.112182]

54. Brabec JL, Lara MK, Tyler AL, Mahoney JM. System-Level Analysis of Alzheimer's Disease Prioritizes Candidate Genes for Neurodegeneration. *Front Genet*. 2021 Apr 6;12:625246. [PMID: 33889174 DOI: 10.3389/fgene.2021.625246]

55. Wu W, Du S, Shi W, et al. Inhibition of Rac1-dependent forgetting alleviates memory deficits in animal models of Alzheimer's disease. *Protein Cell.* 2019 Oct;10(10):745-759. [PMID: 31321704 DOI: 10.1007/s13238-019-0641-0]

56. Kikuchi M, Sekiya M, Hara N, et al. Disruption of a RAC1-centred network is associated with Alzheimer's disease pathology and causes age-dependent neurodegeneration. *Hum Mol Genet*. 2020 Mar 27;29(5):817-833. [PMID: 31942999 DOI: 10.1093/hmg/ddz320]

57. Cai R, Wang Y, Huang Z, et al. Role of RhoA/ROCK signaling in Alzheimer's disease. *Behav Brain Res*. 2021 Sep 24;414:113481. [PMID: 34302876 DOI: 10.1016/j.bbr.2021.113481]

58. Hooper C, Pocock JM. Chromogranin A activates diverse pathways mediating inducible nitric oxide expression and apoptosis in primary microglia. *Neurosci Lett.* 2007 Feb 21;413(3):227-32. [PMID: 17267111 DOI: 10.1016/j.neulet.2006.11.068]

59. Dhawan G, Combs CK. Inhibition of Src kinase activity attenuates amyloid associated microgliosis in a murine model of Alzheimer's disease. *J Neuroinflammation.* 2012 Jul 2;9:117. [PMID: 22673542 DOI: 10.1186/1742-2094-9-117]

60. Lam V, Takechi R, Pallebage-Gamarallage MM, Galloway S, Mamo JC. Colocalisation of plasma derived apo B lipoproteins with cerebral proteoglycans in a transgenic-amyloid model of Alzheimer's disease. *Neurosci Lett.* 2011 Apr 4;492(3):160-4. [PMID: 21310214. DOI: 10.1016/j.neulet.2011.02.001]

61. Fassbender K, Walter S, Kühl S, et al. The LPS receptor (CD14) links innate immunity with Alzheimer's disease. *FASEB J*. 2004 Jan;18(1):203-5. [PMID: 14597556 DOI: 10.1096/fj.03-0364fje]

62. Liu Y, Walter S, Stagi M, et al. LPS receptor (CD14): a receptor for phagocytosis of Alzheimer's amyloid peptide. *Brain.* 2005 Aug;128(Pt 8):1778-89. [PMID: 15857927 DOI: 10.1093/brain/awh531]

63. Reed-Geaghan EG, Reed QW, Cramer PE, Landreth GE. Deletion of CD14 attenuates Alzheimer's disease pathology by influencing the brain's inflammatory milieu. *J Neurosci*. 2010 Nov 17;30(46):15369-73. [PMID: 21084593 DOI: 10.1523/JNEUROSCI.2637-10.2010]

64. Ma D, Fetahu IS, Wang M, et al. The fusiform gyrus exhibits an epigenetic signature for Alzheimer's disease. *Clin Epigenetics*. 2020 Aug 27;12(1):129. [PMID: 32854783 DOI: 10.1186/s13148-020-00916-3]

65. Pang C, Yang H, Hu B, et al. Identification and Analysis of Alzheimer's Candidate Genes by an Amplitude Deviation Algorithm. *J Alzheimers Dis Parkinsonism*. 2019;9(1):460. [PMID: 31080696 DOI: 10.4172/2161-0460.1000460]

66. Cheng JS, Dubal DB, Kim DH, et al. Collagen VI protects neurons against Abeta toxicity. *Nat Neurosci*. 2009 Feb;12(2):119-21. [PMID: 19122666 DOI: 10.1038/nn.2240]

67. Hondius DC, Eigenhuis KN, Morrema THJ, et al. Proteomics analysis identifies new markers associated with capillary cerebral amyloid angiopathy in Alzheimer's disease. *Acta Neuropathol Commun*. 2018 Jun 4;6(1):46. [PMID: 29860944 DOI: 10.1186/s40478-018-0540-2]

68. Snow AD, Mar H, Nochlin D, Kresse H, Wight TN. Peripheral distribution of dermatan sulfate proteoglycans (decorin) in amyloid-containing plaques and their presence in neurofibrillary tangles of Alzheimer's disease. *J Histochem Cytochem*. 1992 Jan;40(1):105-13. [PMID: 1370306 DOI: 10.1177/40.1.1370306]

69. Shioya M, Obayashi S, Tabunoki H, et al. Aberrant microRNA expression in the brains of neurodegenerative diseases: miR-29a decreased in Alzheimer disease brains targets neurone navigator 3. *Neuropathol Appl Neurobiol*. 2010 Jun;36(4):320-30. [PMID: 20202123 DOI: 10.1111/j.1365-2990.2010.01076.x]

70. Romito-DiGiacomo RR, Menegay H, Cicero SA, Herrup K. Effects of Alzheimer's disease on different cortical layers: the role of intrinsic differences in Abeta susceptibility. *J Neurosci*. 2007 Aug 8;27(32):8496-504. [PMID: 17687027 DOI: 10.1523/JNEUROSCI.1008-07.2007]

71. Morimoto H, Wada J, Font B, et al. Procollagen C-proteinase enhancer-1 (PCPE-1) interacts with beta2-microglobulin (beta2-m) and may help initiate beta2-m amyloid fibril formation in connective tissues. *Matrix Biol.* 2008 Apr;27(3):211-9. [PMID: 18164932 DOI: 10.1016/j.matbio.2007.11.005]

72. Salza R, Peysselon F, Chautard E, et al. Extended interaction network of procollagen C-proteinase enhancer-1 in the extracellular matrix. *Biochem J*. 2014 Jan 1;457(1):137-49. [PMID: 24117177 DOI: 10.1042/BJ20130295]

73. Strunz M, Jarrell JT, Cohen DS, Rosin ER, Vanderburg CR, Huang X. Modulation of SPARC/Hevin Proteins in Alzheimer's Disease Brain Injury. *J Alzheimers Dis*. 2019;68(2):695-710. [PMID: 30883351 DOI: 10.3233/JAD-181032]

74. Muraoka S, DeLeo AM, Yang Z, Tatebe H, Yukawa-Takamatsu K, Ikezu S, Tokuda T, Issadore D, Stern RA, Ikezu T. Proteomic Profiling of Extracellular Vesicles Separated from Plasma of Former National Football League Players at Risk for Chronic Traumatic Encephalopathy. Aging Dis. 2021 Sep 1;12(6):1363-1375. [PMID: 34527415 doi: 10.14336/AD.2020.0908]
